# Pulmonary hypertension: Linking inflammation and pulmonary arterial stiffening

**DOI:** 10.3389/fimmu.2022.959209

**Published:** 2022-10-05

**Authors:** Shao-Fei Liu, Netra Nambiar Veetil, Qiuhua Li, Mariya M. Kucherenko, Christoph Knosalla, Wolfgang M. Kuebler

**Affiliations:** ^1^ Institute of Physiology, Charité-Universitätsmedizin Berlin, Corporate Member of Freie Universität Berlin and Humboldt-Universität zu Berlin, Berlin, Germany; ^2^ German Centre for Cardiovascular Research (DZHK), Berlin, Germany; ^3^ Department of Cardiothoracic and Vascular Surgery, German Heart Center, Berlin, Germany; ^4^ Charité-Universitätsmedizin Berlin, Corporate Member of Freie Universität Berlin and Humboldt-Universität zu Berlin, Berlin, Germany; ^5^ German Center for Lung Research (DZL), Gießen, Germany; ^6^ The Keenan Research Centre for Biomedical Science, St. Michael’s Hospital, Toronto, ON, Canada; ^7^ Department of Surgery and Physiology, University of Toronto, Toronto, ON, Canada

**Keywords:** pulmonary hypertension, inflammation, vascular stiffness, vascular calcification, ECM remodeling

## Abstract

Pulmonary hypertension (PH) is a progressive disease that arises from multiple etiologies and ultimately leads to right heart failure as the predominant cause of morbidity and mortality. In patients, distinct inflammatory responses are a prominent feature in different types of PH, and various immunomodulatory interventions have been shown to modulate disease development and progression in animal models. Specifically, PH-associated inflammation comprises infiltration of both innate and adaptive immune cells into the vascular wall of the pulmonary vasculature—specifically in pulmonary vascular lesions—as well as increased levels of cytokines and chemokines in circulating blood and in the perivascular tissue of pulmonary arteries (PAs). Previous studies suggest that altered hemodynamic forces cause lung endothelial dysfunction and, in turn, adherence of immune cells and release of inflammatory mediators, while the resulting perivascular inflammation, in turn, promotes vascular remodeling and the progression of PH. As such, a vicious cycle of endothelial activation, inflammation, and vascular remodeling may develop and drive the disease process. PA stiffening constitutes an emerging research area in PH, with relevance in PH diagnostics, prognostics, and as a therapeutic target. With respect to its prognostic value, PA stiffness rivals the well-established measurement of pulmonary vascular resistance as a predictor of disease outcome. Vascular remodeling of the arterial extracellular matrix (ECM) as well as vascular calcification, smooth muscle cell stiffening, vascular wall thickening, and tissue fibrosis contribute to PA stiffening. While associations between inflammation and vascular stiffening are well-established in systemic vascular diseases such as atherosclerosis or the vascular manifestations of systemic sclerosis, a similar connection between inflammatory processes and PA stiffening has so far not been addressed in the context of PH. In this review, we discuss potential links between inflammation and PA stiffening with a specific focus on vascular calcification and ECM remodeling in PH.

## Introduction

Pulmonary hypertension (PH) comprises a group of diseases in which the mean pulmonary artery pressure (mPAP) exceeds 25 mmHg at rest according to current guidelines (1). Recently, the 6th World Symposium on PH has recommended to lower this cutoff further to 20 mmHg (2). The World Health Organization (WHO) classifies PH into five groups based on identifiable cause and risk factors (3). Although the treatment of pulmonary arterial hypertension (PAH) (WHO Group 1) has entered the stage of targeted therapy, the 5-year survival rate of patients with PAH is still only approximately 50% (4), presumably due to the multifactorial pathophysiological mechanisms of PAH, which evade targeting by a single pharmacological drug, in particular at the advanced disease stage (5). Therefore, identification and therapeutic targeting of common upstream mechanisms that trigger multiple downstream cellular and molecular processes governing pulmonary vascular remodeling in different PH groups remains the ultimate goal for an improved care of PH patients.

**Figure 1:**
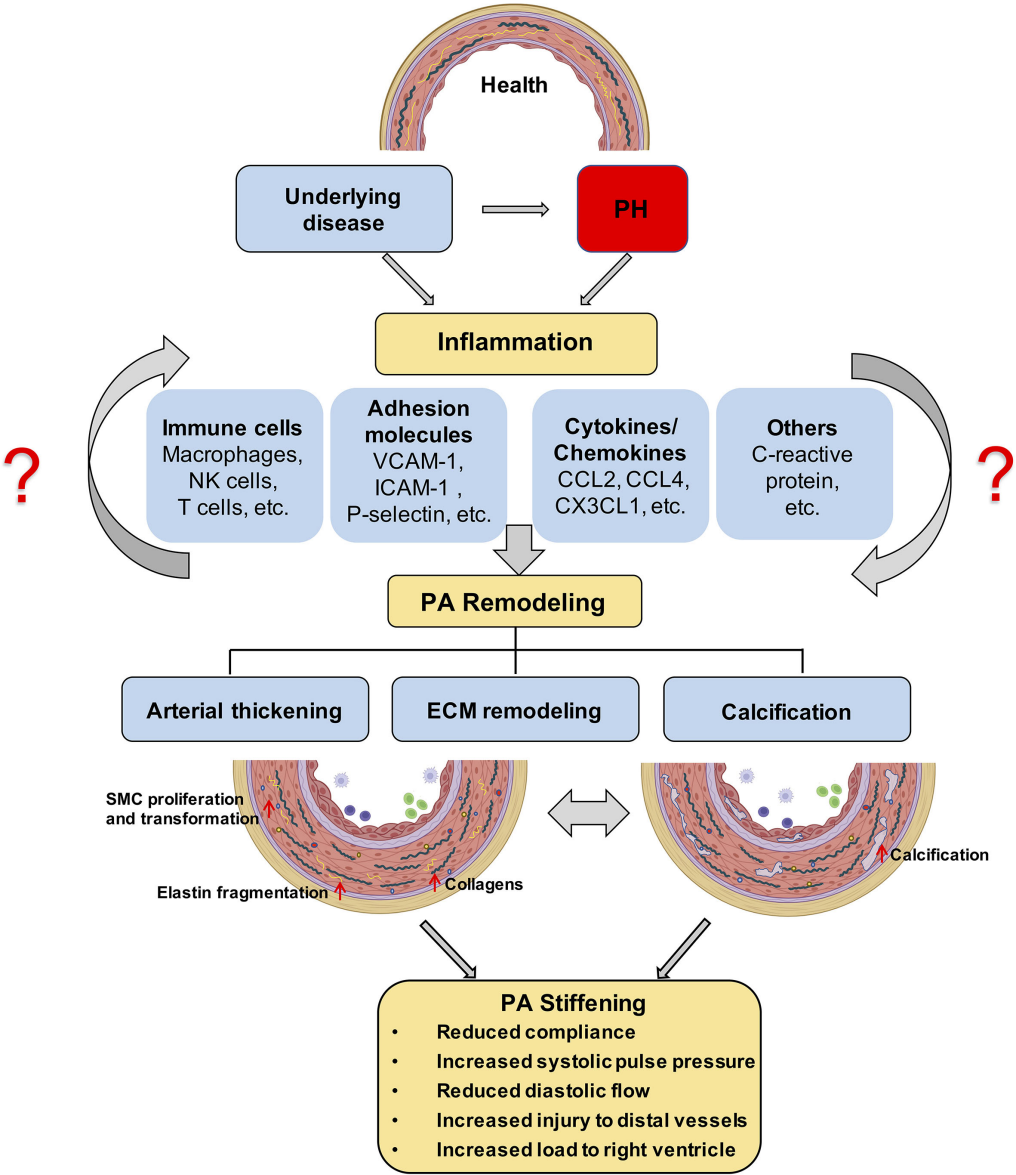
Proposed role of inflammation in PA stiffening; The development of PH is associated with an inflammatory response in the pulmonary vasculature, characterized by immune cell infiltration and the secretion of immune factors. ECM stiffening, especially proximal large pulmonary vascular sclerosis, occurs in the early stages of PH and has a prognostic value for patient outcome and later calcification, and is driven by inflammation. Prolonged angiosclerosis, in turn, further promotes an inflammatory response that exacerbates pulmonary vascular calcification and thickening. PH, pulmonary hypertension; ECM, extracellular matrix; PA, pulmonary artery.

Lately, pulmonary perivascular inflammation has gradually gained increased attention as an early common hallmark across different PH groups. In the early stage of the disease, PAH patients and corresponding animal models not only display an accumulation of immune cells such as macrophages (6, 7) and mast cells (8) in their lungs (9), but also have elevated levels of inflammatory mediators in their pulmonary circulation (10, 11) (**Figure 1**). In most forms of PH, this inflammatory response is predominantly localized to the pulmonary adventitia (7). In fact, changes in the adventitia, which consists of a complex mix of heterogeneous cells, tend to precede those in other vascular compartments and are required for vascular remodeling (12). In PAH, this spatial predilection has been linked to the fact that fibroblasts in the pulmonary adventitia exhibit a pro-inflammatory phenotype with an increased expression of inflammatory mediators that drive the recruitment of innate immune cells (7, 13, 14). The resulting perivascular inflammation is now considered to constitute a critical pathomechanism orchestrating remodeling from the outside-in not only in PH associated with disorders of the immune system, such as connective tissue disease-associated pulmonary arterial hypertension (CTD-PAH) (15), but also in other forms of PAH (11, 16) as well as in PH due to left heart disease (PH-LHD) (17). In parallel, the adventitia releases a myriad of factors that regulate differentiation, proliferation, apoptosis, migration, and collagen synthesis by other cells in the vessel wall, while adventitial fibroblasts can transform to myofibroblasts and migrate into the intima through the medial layer (12). As such, it has been proposed that inflammatory processes alter vascular and immune cell metabolism, ultimately enhancing pulmonary artery (PA) remodeling and aggravating PH (**Figure 1**).

Concomitantly over the past decade, PA stiffening has emerged as an early hallmark, pathomechanism, and predictor of morbidity and mortality in PH (18–20). Vascular stiffening, defined as increased resistance of the arterial wall to deformation during blood influx, is a consequence of pathological vascular remodeling that can occur in both large proximal arteries and small distal arteries and arterioles. The mechanical consequences of these structural changes are decreased compliance in proximal PAs, and increased resistance to blood flow (pulmonary vascular resistance, PVR) in distal PAs (21). PA compliance (PAC) is essential to transform the pulsatile blood flow that enters the large conduit arteries *via* the Windkessel effect into the nearly laminar flow at the level of the distal pulmonary vascular tree. As such, PAC reduces right ventricular (RV) afterload and maintains near-constant lung perfusion over the cardiac cycle. In line with the impact of PAC for RV function, invasive or non-invasive assessment of PAC (or capacitance) has revealed PA stiffening in PAH patients as a sensitive predictor of pathological RV remodeling and mortality (21–24). It has further been proposed that stiffening of proximal PAs, through elevation of pulse-wave velocity and the shear stress exerted by the blood, promotes injury and remodeling in distal vessels, thus driving the pathology of PH in a positive feed-forward loop (25). Such interdependency between proximal and distal PA regions would predict that pathological remodeling should occur in parallel in large and small vessels. Indeed, work by Stuart R. Reuben first identified a hyperbolic relationship between PAC and PVR (26). The product of PAC × PVR yields the resistance–compliance (RC) time, which is considered to remain almost constant in PH patients of WHO class I (PAH), III (PH due to chronic lung disease), IV (chronic thrombo-embolic PH), or V (PH with unclear multifactorial mechanisms) and independent of medical therapy (27). Interestingly, however, for patients with WHO class II PH (PH due to left heart disease), RC time is reduced, i.e., for any given PVR, the corresponding PAC is lower as compared to PH patients from other causes. Notably, this reduction in RC time is also associated with an increase in RV afterload (27). This interesting finding may indicate distinct pathomechanisms and/or a higher degree of stiffening in proximal PAs in PH patients with underlying left heart disease as compared to other forms of PH; yet, this notion remains to be rigorously tested and mechanistically explored.

Conversely, mechanical communication between proximal PAs and the distal pulmonary vasculature may also promote restoration of pulmonary vascular homeostasis. Evidence of such a reverse remodeling process derives from a few clinical studies in patients with congenital heart disease and PH due to intracardiac left-to-right shunts causing lung overperfusion. In these patients, surgical banding of the PA—performed with the intent to protect the proximal PA from excessive pressure and flow—could successfully improve PH and, in some cases, reverse vascular remodeling in distal arteries (28, 29).

A growing number of studies reporting techniques to estimate stiffness of proximal PAs *in vivo* show promise for the use of PA stiffness estimates as a prognostic tool in PH. Most commonly, PA stiffness is estimated by calculation of pulmonary arterial capacitance as ratio of stroke volume over pulmonary pulse pressure, assessed by either cardiac catheterization or non-invasively by echocardiography (20, 30–37), or by calculation of a stiffness index as change in PA pressure (again assessed by right heart catheterization) divided by the corresponding change in PA diameter (determined by real-time imaging modalities, such as cardiac magnetic resonance imaging) (18, 38).

Artery stiffening in cardiovascular disease is mainly attributed to remodeling of the extracellular matrix (ECM) and/or calcification within the arterial wall (39–42) (**Figure 1**). In particular, PAH is characterized by remodeling of the ECM and thickening of all three layers of the PA wall (43), which ultimately reduces arterial compliance. PAs of PAH patients exhibit an increased deposition of interstitial collagen, including collagen I, collagen XIV, and basement membrane-specific collagens, especially collagen IV (43–45). Additionally, increased expression of other ECM proteins such as elastin and fibronectin, or the matricellular ECM protein tenascin-C by dedifferentiated adventitial fibroblasts has been reported in PAH patients (46). Increased production and deposition of ECM constituents in PAs is considered to occur as an adaptive response to increased digestion of medial and basement membrane (BM) ECM by matrix metalloproteinases (MMPs), which have been found to be increased in PAH (47) and IPAH patients (45). The elevated expression of collagens by endothelial cells (ECs), smooth muscle cells (SMCs), and adventitial fibroblasts is associated with increased collagen-cross-linking by lysyl oxidases (LOXs) (48). In addition, proteolytic enzymes also induce degradation of elastic fibers, which are challenging to rebuild despite increased elastin gene expression due to the multicomponent complex 3D structure of these fibers (49–53). As such, PA stiffening emerges as a progressive imbalance of collagen over elastin fiber components in the PA wall.

Vascular stiffening has also been attributed to vascular calcification (40), a pathological deposition of solid minerals within the intima or media of arterial walls (54) (**Figure 1**). Importantly, pulmonary vascular calcification has been associated with transdifferentiation of SMCs into osteogenic-like lineages, driven by the activity of the pro-osteogenic transcription factor Runt-related transcription factor 2 (RUNX2) (55). As such, increased nuclear expression of RUNX2 in PA SMCs not only activates expression of calcification-related biomineralization genes (56), but also promotes cell proliferation and resistance to apoptosis by activating hypoxia-inducible factor-1α (HIF-1α) (55).

Stiffening of proximal PAs in PAH patients (18, 57) increases pulse pressure and shear stress in the pulmonary vasculature. Of relevance, these alterations in biomechanical forces acting upon the lung vascular wall can induce pro-inflammatory responses in ECs of distal PAs (58, 59) and promote the aggregation of immune cells (58). This includes inflammatory cell recruitment and release of immune-cell-derived cytokines, such as IL-6 (60, 61) and TNF (62) and bioactive enzymes, including MMPs (46), which may, in turn, promote vascular remodeling and stiffening processes, thus establishing a progressive vicious cycle. Such interplay between inflammation-triggered signaling events that, in turn, initiate wound healing processes and ECM remodeling, ultimately culminating in tissue fibrosis and scar formation, is well established in cardiac and systemic vascular diseases (63–65). In PH, however, the cause–effect relationship between inflammatory signaling and vascular stiffening has so far neither clinically nor experimentally been addressed. As such, the present review aims to link known inflammatory responses in PH to processes related to vascular stiffening, namely, ECM remodeling and vascular calcification, identified in either PH or other vascular diseases and *vice versa.* Proposed links and relevant literature are summarized in **Table 1** and will be discussed in detail below. As such, we intend to highlight the potential relevance of a pathophysiological axis between inflammation and PA stiffening, and to incite mechanistic studies to address this conceptual gap in our present understanding of PH.

**Table 1:** Inflammatory mediators associated with vascular stiffening.

Cytokines, immune cells, and adhesion molecules in PH					Regulation of vascular stiffening-related pathways
Category/name		Cell/tissue type of increased mediator abundance in PH patients/animal models	WHO-defined PH group		
**Cytokines**	IL-1β	Lung (66–69), Plasma (70), Fibroblasts (71), CTEPH-EC (72)	I (68, 69, 71), III (66, 67, 70), IV (72)		**Atherosclerosis** IL-1β is associated with calcium content and calcification of the aortic wall (73). **Cardiovascular disease** IL-1β and TGF-β initiate the transdifferentiation of cardiac fibroblasts to myofibroblasts that produce elevated levels of collagens after cardiac injury (74). **Aortic calcification** IL-1β and TNF modulate EndoMT of aortic ECs and make ECs more sensitive to osteogenic transdifferentiation by BMP-9 *in vitro*, predominantly by reducing BMPR2 expression and increasing JNK signaling (75).
	IL-2	Plasma (76)	I (76)		**Aortic stiffening** In mice, IL-2 reduces angiotensin II-mediated inflammation and aortic stiffening *via* activation of CD4^+^CD25^+^Foxp3^+^ regulatory T cells (77).
	IL-6	Plasma (70, 76, 78–80), Lung (61, 66–69, 81–83), Serum (16, 61, 84, 85), SMC (84), Pulmonary veins (61), PA (61), Exhaled breath condensate (85), Fibroblast (71)	I (16, 68, 69, 71, 76, 79, 80, 83, 84), II (61, 81), III (66, 67, 70, 78, 82, 85)		**IL-6 in PH-LHD** In a rat model of PH-LHD, macrophage accumulation and increased IL-6 production were observed in the lung (8, 81). IL-6 activates STAT3 signaling, inducing PA SMC overproliferation (81). **IL-6 and calcification in PAH** MicroRNA-204 regulates BRD4 expression, which upregulates IL-6 and drives vascular calcification in PAH (86, 87). **Coronary artery disease (CAD)** CAD patients have increased osteoprotegerin, osteopontin, and IL-6 levels in serum (88).
					**Hypertension-induced aortic stiffening** Positive correlation between IL-6 and aortic stiffness (89) **Arterial stiffening in chronic kidney disease (CKD)** IL-6 levels in patient plasma are positively correlated to arterial wall stiffness (90). **Vascular remodeling in PH** IL-6 promotes SMC proliferation and migration in PH, leading to medial wall thickening in distal PAs (60). IL-6 upregulates MMP-expression in PH, promoting ECM remodeling (60). IL-6 depletion attenuates lung vascular remodeling in a rat MCT model of PH (8).
	IL-10	Plasma (79, 80, 91), Lung (69)	I (69, 79, 80), IV (91)		**Aortic stiffness** IL-10 knockout mice develop aortic stiffening due to increased COX-2 activity and resulting thromboxane A2 receptor activation (92).
	IL-12	Plasma (79, 93), Serum (94, 95)	I (79, 93–95)		**Atherosclerotic cardiovascular disease** In CVD patients, IL-12 serum levels positively correlate with arterial stiffness (96).
	IL-17	Lung (83), Plasma (79), CD4+T cell (97)	I (79, 83), III (97)		**Psoriasis** IL-17 increases aortic stiffness by reducing lipoprotein trafficking (98).
	TNF	Plasma (70, 99–101), Lung (61), Serum (61, 102), Pulmonary veins (61), PA (61), EC (103)		I (100, 101), II (61), III (70), IV (99, 102)	**Aortic calcification** TNF induces osteoblast markers and enhanced osteoblast differentiation and calcification in bovine aortic SMCs by activation of the cAMP pathway (104). **Psoriasis** The anti-TNF monoclonal antibody adalimumab reduces carotid arterial stiffness (105). **Estrogen deficiency in postmenopausal women** The TNF inhibitor etanercept reduces carotid arterial stiffness (106). **Inflammatory artheropathies** In a controlled clinical study, patients with rheumatoid arthritis, ankolysing spondylitis, and psoriatic arthritis that received anti-TNF therapies (either adalimumab, ethanarcept, or infliximab) exhibited less aortic stiffness, assessed by aortic pulse wave velocity and augmentation index (107)
	IL-4 IL-7 IL-8 IL-13 IL-18 IL-21 IL-33 IFN-γ	Lung (108), Plasma (79, 109) Plasma (79) Exhaled breath condensate (85), Plasma (79, 80), EC (72, 103) Lung (108), Plasma (109) Lung (66, 110) Lung (82) Lung (111, 112), Serum (113) Plasma (76, 109)		I (79, 108), III (109) I (79) I (79), III (85), IV (72, 80) I (108), III (109) III (66, 110) III (82) I (111, 112), III (113) I (76), III (109)	Not studied in the context of vascular stiffening
	CCL2 (MCP-1)/CCR2	Lung (114), SMC (115), Macrophage (115, 116), Fibroblast (71), CTEPH-EC (72), Plasma (91, 99)		I (71, 115, 116), III (114), IV (72, 91, 99)	**Hypertension-induced aortic stiffness** Positive correlation between MCP-1 levels in patient plasma and aortic stiffness estimated by echocardiography (89). **Arterial stiffening in chronic kidney disease (CKD)** Positive correlation between angiopoietin-2 in serum of CKD patients and aortic stiffness. Angiopoietin-2 induces CCL2 in ECs (117)
	CCL7/CCR7	Plasma (79), Serum (94), Fibroblast (71)		I (71, 79, 94)	**Abdominal aortic stiffness** HIF-1α deficiency in vascular smooth muscle cells suppresses CCL7, which increases macrophage infiltration (118).
	CX3CL1/CX3CR1 CCL4 CCL5 (RANTES)/CCR5 CCL11 CCL12 (SDF-1) CXCR1 CXCR4/CXCL12 CXCL9 CXCL13 CD40	Lung (114), Serum (94) Plasma (79) PAEC (119), Plasma (79), PASMC (115, 120), Macrophages (115), Fibroblasts (71), Lung (121), CTEPH-EC (72), PAH-EC (122) Plasma (79) Fibroblasts (71), Lung (121) Lung (121) Fibroblasts (71), Lung (123, 124) Plasma (80) Plasma (80), Serum (125) Fibroblasts (71), Serum (126), Lung (127)		I (94), III (114) I (79) I (71, 79, 115, 119–122), III (120), IV (72, 91, 99) I (79) I (71, 121) I (121) I (71, 123), III (123, 124) I (80), IV (80) I (80, 125), IV (125) I (71), III (126) I, III (127)	Not studied in the context of vascular stiffening
**Immune cells**	Macrophages	Bone marrow (128), Lung (81, 129, 130), CTEPH-EC (131, 132), Alveoli (128), Blood (133)		I (115, 128, 129, 133, 134), II (81), III (130), IV (131, 132)	**PAH** Infiltrated macrophages express MMP-10, resulting in ECM remodeling and PA stiffening (47). **Thoracic aorta stiffening in CKD** ETA receptor blockade reduces macrophage infiltration, aortic stiffness and calcification in rats (135). **Aortic stiffness in obesity** Peroxisome proliferator-activated receptor γ (PPARγ) activation by pioglitazone attenuates MMP-12 in macrophages *in vitro*, and reduces aortic stiffness *in vivo* (136). **Aortic stiffness in abdominal aortic aneurysm** Angiotensin II promotes the recruitment of M2-like macrophages in the aorta of IL12p40-deficient mice, which promote medial remodeling and aortic stiffening through increased TGF-β production (137).
	CD4^+^CD25^+^Foxp3^+^ regulatory T cells	Plasma (76)		I (76, 138), III (139)	**Aortic stiffening** *In vivo* CD4^+^CD25^+^Foxp3^+^ regulatory T-cell stimulation in mice reduces angiotensin-II mediated aortic remodeling and stiffening (77).
	NK cells	Plasma (76), CTEPH-EC (132), Blood (140)		I (76, 140), IV (132)	**PA calcification** Granzyme B from nature killer cells increases calcification in smooth muscle cells (SMCs) under hypoxia (141)
	T cells	Plasma (76) Lung (142)		I (76), III (142)	**HIV-related arterial stiffening** CD4^+^ and CD8^+^ T-cell exhaustion is associated with arterial stiffness (143).
	Neutrophil cells	Blood/bone marrow (144)		I (144), III (144)	**Vasculature stiffening** Oxidized low-density lipoprotein (OxLDL) and stiffer substrates promote neutrophil transmigration *in vitro* (145)
	Mast cells B cells Dendritic cells Eosinophils	Lung (8, 146–148), CTEPH-EC (132), Blood (149) Lung (8) Lung (69) Lung (150)		I (8, 147–149), II (8, 146), III (8), IV (132) I (8) I (69) I (150)	Not studied in the context of vascular stiffening
**Other mediators**	C-reactive protein (CRP)	Plasma (91, 99, 116)		I (116), IV (91, 99)	**Arterial stiffening** Higher CPR levels are associated with increased arterial stiffness (151).
	Intercellular adhesion molecule-1 (ICAM-1)	Plasma (93)		I (93)	**Arterial stiffening in CKD** Plasma angiopoietin-2, which induces ICAM-1 in ECs (117), correlates with arterial stiffness in CKD. **Matrix stiffness** Stiff matrices induce ICAM-1 clustering in ECs, which promotes immune cell recruitment (152).
	Vascular cell adhesion molecule-1 (VCAM-1)	Plasma (93), Fibroblasts (71), Lung (121), CTEPH-EC (72)		I (71, 93, 121), IV (72)	**Atherosclerosis** MicroRNA-1185 correlates with arterial stiffness and VCAM-1 expression (153).
	Macrophage inflammatory protein-1α	Plasma (91)		IV (91)	Not studied in the context of vascular stiffening

## Inflammation-induced arterial wall thickening and ECM remodeling

PA stiffening and inflammatory responses are both paramount characteristics of PH. While inflammation is commonly associated with PH in both animal models and clinical scenarios, little is known about the role of inflammation in inducing vascular remodeling in PH. Only a limited number of studies have so far addressed the role of inflammation in promoting the production of ECM components (154), namely, collagens (155), fibronectin (156), and tenascin-C (156) in PH. Yet, in other cardiovascular diseases, the connection between inflammation and increased vascular stiffness has been better characterized: here, inflammatory processes have been shown to promote arterial stiffening through a variety of mechanisms, including the induction of endothelial dysfunction and BM stiffening, increased proliferation of SMCs (49)—resulting in arterial wall thickening and reduced compliance—and remodeling and stiffening of the ECM in different segments of the arterial wall.

In PH, elevated pressure and high pulsatile flow as a consequence of reduced vascular compliance can be sensed by ECs of the pulmonary vascular bed. Specifically in hypoxia-induced PH, ECs produce elevated levels of the inflammatory cytokines IL-1β (9) and IL-6 (9, 60), and express increased levels of immune cell adhesion molecules including vascular cell adhesion molecule-1 (VCAM-1), intercellular adhesion molecule-1 (ICAM-1), and P-selectin (9). Concomitantly, other vascular resident cells, such as SMCs and fibroblasts, respond to biomechanical cues by altered secretion of immune factors including inflammatory cytokines such as monocyte chemoattractant protein-1 (MCP-1), stromal cell-derived factor 1, and CCR5 (71) (**Table 1**). These inflammatory mediators can, in turn, induce PA remodeling and stiffening (9, 71, 157). While the links between increased inflammation and PA remodeling are so far little understood in PH, we will delineate in the following existing connections between key inflammatory signals and vascular stiffening in systemic cardiovascular diseases, with the aim to translate this knowledge into an advanced understanding of the potential role of inflammation in PA stiffening in PH.

**Figure 2:**
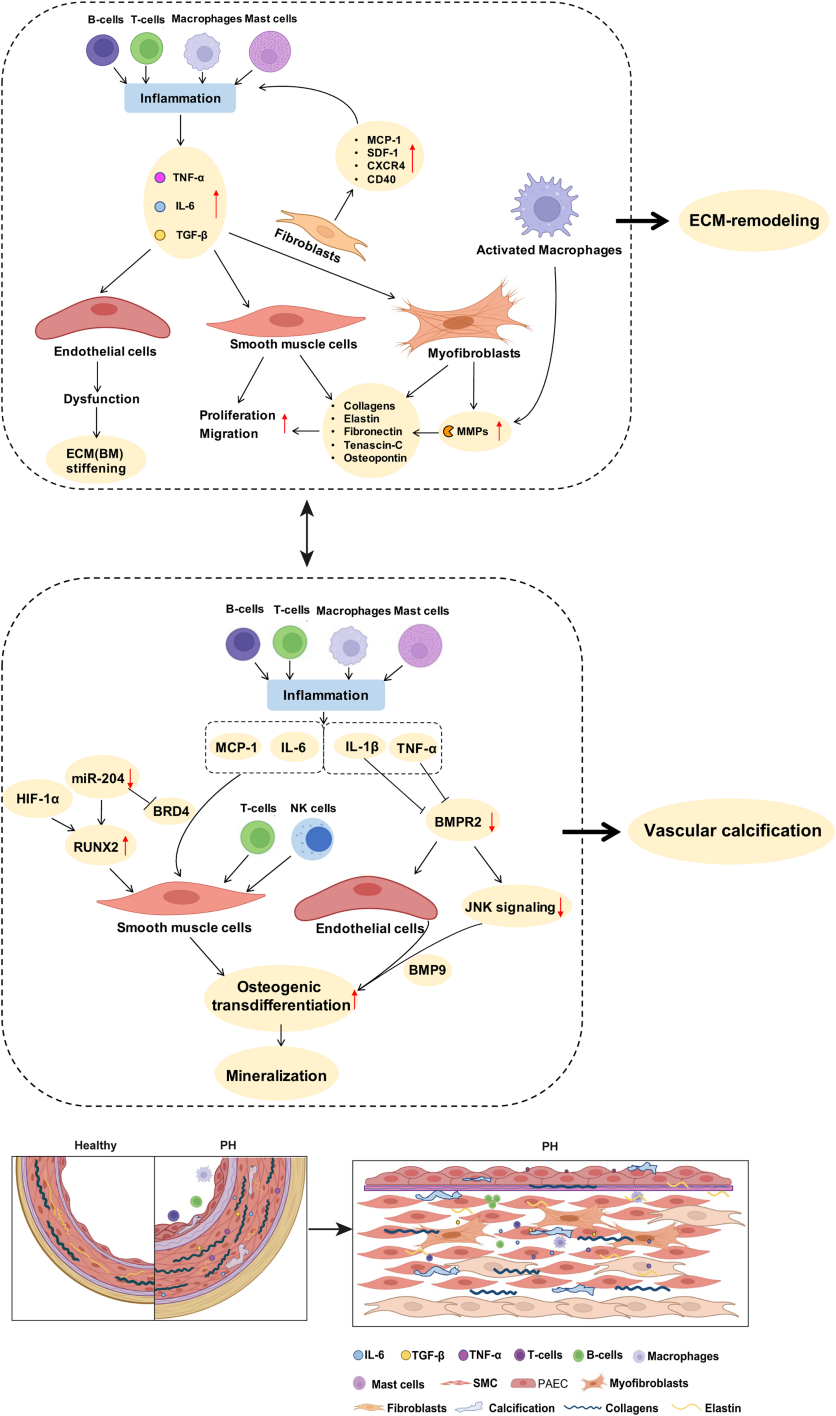
Potential links between inflammatory mediators and mechanisms of pulmonary arterial ECM remodeling and vascular calcification in PH. As described in detail in the manuscript text, perivascular accumulation of immune cells is a characteristic feature of PH. Inflammatory cells such as macrophages produce MMPs that promote ECM degradation and remodeling. Inflammatory cytokines such as IL-6 and TGF-β drive the proliferation of PA SMCs. Stimulation of fibroblasts by inflammatory mediators increases the expression of collagens, elastin, and fibronectin, further promoting PA stiffness. Activated immune cells and inflammatory mediators promote SMC transdifferentiation and enhance the expression of biomineralization genes, thus driving vascular calcification. BMPR2 downregulation, especially in response to the inflammatory factor TNF, promotes endothelial cell mesenchymalization and may as such contribute to the development of pulmonary vascular calcification. A detailed discussion of the proposed signaling pathways is provided in the manuscript text. ECM, extracellular matrix; MMPs, matrix metalloproteinases; SMC, smooth muscle cells; PH, pulmonary hypertension; PA, pulmonary artery; BM, basement membrane.

Several key inflammatory signals induce PA remodeling by dysregulating the behavior and function of both ECs and SMCs in PH, ultimately leading to arterial wall thickening and stiffening. Among these, IL-6 and TNF were found to be increased in plasma, lung, pulmonary arteries and veins, as well as in PA ECs in both patients and animal models of various PH groups (**Table 1**). In PAH patients (60) and in PH-LHD rat models (61, 81), IL-6 contributes to PA remodeling by inducing medial wall thickening *via* SMC proliferation and muscularization of the distal pulmonary arterial tree due to migration of SMCs into precapillary arterioles (60, 61, 81) (**Table 2**; **Figure 2**), potentially affecting arterial compliance by increased wall thickening. In the pulmonary adventitia, fibroblasts activate recruited macrophages through paracrine IL-6 signaling, initiating a pro-inflammatory and pro-fibrotic phenotype that is associated with an increased inflammatory response and vascular remodeling in PH (7). Notably, IL-6 is a sensitive marker for systemic inflammation in cardiovascular disease (60, 88). In rheumatoid arthritis (164) and acute ischemic stroke (165), elevated levels of IL-6 in patient serum were associated with aortic stiffening as estimated by pulse-wave velocity, which could be significantly reduced by therapeutic infusions of the anti-IL-6 receptor antibody tocilizumab (164).

**Table 2:** Potential links between factors associated with PA stiffening and immune responses in PH.

Factors associated with PA stiffening	Potential link to immune responses in PH
Caveolin-knockout mice show increased PA stiffness (158).	Caveolin-1 inhibits adventitial macrophage-induced inflammation in mouse aortic vessels (159).
5-HT inhibition prevents hypoxia-induced PH and vascular remodeling of PAs in mice (160).	5-HT is widely expressed on immune cells such as dendritic cells, and triggers the release of IL-1 and IL-6 (161).
SMC overproliferation causes arterial thickening and distal PA muscularization leading to arterial stiffening in PH mice (60).	IL-6 overexpression in inflammation triggers SMC hypertrophy in PAs (60).
MMP-overexpression and activation lead to degradation of elastin fibers in the PA wall and arterial stiffening in PAH patients (162).	Activated macrophages secrete MMP-2 (162), MMP-9 (162), MMP-10 (47), and MMP-19 (6, 154) in PAH. IL-6 upregulates MMP-9 expression in SMCs in PAH (60).
Myofibroblasts in PH overexpress ECM components (i.e., collagens, fibronectin, tenascin-C, etc.) (163).	TGF-β increases collagen, fibronectin, and tenascin-C production by SMCs and fibroblasts (43). IL-6 and TGF-β induce differentiation of fibroblasts to myofibroblasts (21, 46, 71).

Similarly, elevated TNF in rodent models of PAH and PH-LHD has been shown to result in increased PA EC and SMC proliferation and medial wall thickening (61, 166), which have been attributed to suppressed BMPR-II signaling in PAH (166). Due to its effects on SMC hyperplasia, TNF may also promote PA stiffening in PH; however, direct correlations between TNF levels and PA stiffness in PH have yet to be established. In other cardiovascular and inflammatory diseases, e.g., arteriosclerosis, TNF is an established key mediator of vascular remodeling (61, 62). Patients with inflammatory artheropathies, namely, rheumatoid arthritis, ankylosing spondylitis, and psoriatic arthritis, who received anti-TNF treatment with either adalimumab, etanercept, or infliximab, showed a reduction in aortic stiffness as assessed by pulse-wave velocity and augmentation index as compared to untreated controls (107, 167). Hence, pharmacological inhibition of inflammatory mediators such as IL-6 and TNF in PH could potentially reduce pulmonary vascular cell proliferation and PA thickening and may therefore present a targeted therapy for PA stiffening.

Furthermore, pro-inflammatory mediators can induce vascular stiffening in cardiovascular diseases by increased production of ECM components, namely, fibrillar and non-fibrillar collagens and fibronectin by resident vascular cells (168). After myocardial infarction as well as in ischemic and non-ischemic heart failure, pro-inflammatory mediators such as TGF-β (74, 169) and IL-1β (170) induce the conversion of fibroblasts into myofibroblasts, which can produce abundant ECM proteins (168) (**Tables 1**, **2**; **Figure 2**). In PH, adventitial myofibroblasts contribute to PA remodeling and stiffening (46) *via* the production of structural ECM components such as collagens, elastin, fibronectin, and dynamic ECM constituents, including tenascin-C and osteopontin (43, 46, 74) (**Table 2**; **Figure 2**). Tenascin-C and osteopontin, in turn, increase fibroblast and SMC proliferation, contributing to myofibroblast conversion and medial thickening, and therefore vascular stiffening (43, 46, 171) (**Figure 2**). Activated macrophages recruited to the pulmonary adventitia may express ECM proteins such as collagen type I, thereby contributing to ECM stiffening in PH (172). In animal models of MCT-induced PH, NADPH oxidase 4 (Nox4) has also been found to be upregulated in the pulmonary adventitia, where it promotes TGF-β-mediated expression of matrix collagens by adventitial fibroblasts and, as such, ECM stiffening (172). Similarly, collagen deposition by resident fibroblasts into the adventitia was also found to be increased in an animal model of chronic hypoxic PH and resulted in a thicker and stiffer arterial wall (172–174). In order to form insoluble rigid fibers, excessive fibrillar collagens are then further cross-linked by cross-linking enzymes (43, 175). Specifically, elevated expression of LOX in SMCs and lysyl oxidase-like enzyme (LOXL) expression in adventitial fibroblasts leads to increased collagen cross-linking and PA stiffening in PAH (176). Moreover, adventitial fibroblasts *per se* exhibit a pro-inflammatory phenotype in PH, including the recruitment and activation of adventitial macrophages (7) and production of pro-inflammatory markers, such as the chemokines MCP-1, SDF-1, RANTES/CCR5, CCR7, CXCR4, and the co-stimulatory molecules CD40 and CD40L (7, 71). This secretory activity can, in turn, create another feedback loop that triggers further inflammation and, hence, ECM remodeling.

Apart from elevated levels of circulating inflammatory mediators, increased mPAP in PH also induces activation of the pro-inflammatory NF-κB signaling pathway in PA ECs and SMCs (58, 133, 157) (**Figure 2**). Based on studies in systemic cardiovascular diseases, such activation of NF-κB emerges as a potentially important step in PA stiffening. As such, nuclear NF-κB was shown to increase the expression of aortic collagen type I in a murine model of type 2 diabetes, resulting in aortic stiffening as measured *ex vivo* by pressure myography (177). Interestingly, these effects were mediated by an NF-κB-dependent overexpression of RUNX2, a key transcription factor relevant not only for ECM remodeling [through increased expression of ECM collagens by SMCs (177)], but also in the context of vascular calcification (55, 177) (as discussed below) (**Figure 2**). It may be speculated that activation of NF-κB could exhibit similar effects in PH, thus contributing to PA stiffening through ECM remodeling and vascular calcification.

Inflammation-induced overproduction of ECM components in cardiovascular diseases is rivaled by elevated proteolytic ECM degradation *via* a parallel increase in MMPs (46). In PH, activated macrophages and myofibroblasts in the adventitia secrete MMPs, specifically MMP-2 (154, 162), MMP-9 (6, 162), MMP-10 (47), and MMP-19 (6, 154), while tissue inhibitors of metalloproteinases (TIMPs) appear downregulated (46, 162) (**Table 2**; **Figure 2**). MMP-2 (50) and MMP-9 (49) degrade elastin, thereby decreasing vessel compliance, resulting in arterial stiffening (49–52). Furthermore, degradation of elastic fibers and other ECM components such as BM collagens, interstitial collagens, fibronectin, and several proteoglycans by MMPs, facilitates migration of adventitial fibroblasts and myofibroblasts into the media and intima, which, in turn, promotes PA stiffening and vascular stenosis (46, 154) (**Table 2**). Similarly, neointimal formation *via* increased proliferation and migration of SMC from the media into the intimal regions of the arterial wall is likewise facilitated by MMP-regulated ECM degradation (52, 178) and promotes vascular stenosis and stiffening (178). Products of ECM proteolysis—the matrikines [recently reviewed in detail by Mutgan et al. (179)]—can, in turn, serve as pro-inflammatory mediators, which accentuate inflammation and may, as such, create another positive feedback loop (43). Furthermore, ECM degradation allows for circulating serum factors to enter the media and stimulate serine elastase production by SMCs (178). These serine elastases aid elastin degradation and the release of activated growth factors, such as fibroblast growth factor (FGF) and TGF-β that, in turn, increase collagen, fibronectin, and tenascin-C production by SMCs and fibroblasts (43)—again furthering PA stiffening (**Table 2**). In other cardiovascular diseases such as ischemic heart failure, immune cells like macrophages, lymphocytes, and mast cells secrete MMPs that remodel the vascular and cardiac ECM in response to mechanical stress (168). In arteriosclerosis, elevated levels of both MMP-2 and MMP-9 were associated with increased arterial stiffness and cardiovascular disease risk, which has been attributed to their ability to degrade the elastic laminae in arteries (180, 181). Accordingly, MMP-2 knockdown reduces arterial stiffening of carotid arteries in mice by decreasing elastin degradation in the tissue (182).

As such, activation of immune cells and inflammatory pathways, and arterial wall thickening and ECM remodeling may reciprocally stimulate each other. Targeting inflammatory processes in cardiovascular diseases, for example, aortic aneurysms, has shown beneficial effects on key mechanisms of ECM remodeling such as elastin degradation, MMP expression, and macrophage infiltration (183). As such, a better understanding of the specific players and molecular pathways involved in this mutual interaction may reveal novel and potentially personalized targets for future PH therapy.

## Pulmonary arterial calcification and inflammation

Biologically induced mineralization is an integral part of human physiology and tissue homeostasis that involves extracellular and intracellular mechanisms to direct the nucleation, growth, and location of the deposited minerals. In disease conditions, these processes may become dysbalanced due to changes in the local or global calcium milieu, DNA damage, endoplasmic reticulum stress, oxidative stress, or metabolic disorders—i.e., processes that are frequently associated with inflammatory responses—and ultimately result in pathological tissue or blood vessel calcification (184, 185). Mechanistically, these factors lead to (or are accompanied by) phenotypic conversion of various cell types into osteoprogenitor cells *via de novo* or increased expression, respectively, of the potent transcription activator RUNX2, which triggers the expression of downstream calcification-promoting proteins such as alkaline phosphatase (186–188). In comparison to systemic arteries, vascular calcification of the PA is scarcely addressed, yet it is actually a common feature in patients with severe prolonged PH (189), advanced PH, and PH with chronic renal failure (190) or end-stage renal disease (191). In fact, detection of peripheral PA calcification by computed tomography (CT) (192) predicts long-term outcome in PH (193) and in patients with atrial septal defect and Eisenmenger’s syndrome (194).

In the context of PAH, a critical role in the regulation of PA calcification has been attributed to a microRNA-204-dependent upregulation of RUNX2 that, in turn, activates HIF-1α, leading to PA SMC hyperproliferation, resistance to apoptosis, and subsequent transdifferentiation into osteoblast-like cells (55). A second study reported that hypoxia-induced circular RNA CDR1 promotes osteogenic transdifferentiation of human PA SMCs by sponging microRNA-7-5p, and consequently upregulating its downstream targets calcium/calmodulin-dependent kinase II-delta (CAMK2D) and calponin 3 (CNN3) (195). Third, PA calcification has been linked to hypoxia, in that hypoxia decreases the expression of serine protease granzyme B stored in the granules of T lymphocytes and natural killer cells, which inhibits store-operated calcium channels (SOCCs) as the main source of calcium mineral by attenuating non-canonical Wnt signals in SMCs, thus increasing calcification of the PA (141). Independent of the underlying pathway, calcification ultimately increases vascular stiffness and reduces the compliance of the pulmonary arterial wall, which is a manifestation of poor prognosis in PH (21).

In the systemic vasculature, inflammatory signals—as seen in PH—have been shown to regulate vascular calcification processes. Specifically, TNF promotes osteogenic differentiation and calcification of bovine aortic SMCs by inducing the expression of osteoblast markers, such as osteoblast-specific factor 2 (Osf2), activator protein 1 (AP1), and cAMP-responsive element-binding protein (CREB) *via* activation of cAMP signaling (104). Likewise, treatment of aortic SMCs with IL-1β or IL-6 caused a dose-dependent increase in alkaline phosphatase activity and increased cell mineralization *in vitro* (196). Interestingly, expression of the inflammatory cytokines IL-6, TNF, and MCP-1 is epigenetically regulated in various tissues by bromodomain protein 4 (BRD4) (86), which modulates the chromatin landscape and activates gene expression by scaffolding transcription factors at gene promoters and/or superenhancers. Notably, BRD4 is upregulated in PA SMCs of PAH patients and in lungs or distal PAs of rat PH models, and is posttranscriptionally regulated by microRNA-204 (87), which is concomitantly involved in PA calcification (55), providing for an additional epigenetic link between inflammation and vascular calcification. More importantly, the RUNX2 gene promoter has been shown to be under direct control of BRD4 during osteoblast differentiation (197) as well as in cancer (198), suggesting that BRD4 may serve as a “master-regulator” of both inflammation and vascular calcification in parallel. In line with this notion, BRD4 inhibition attenuated pulmonary and coronary artery remodeling in experimental PH, and this protective effect was associated with reduced levels of IL-6 and MCP-1 (199, 200).

Although studies linking calcification and inflammation in PH are scarce, cytokines have been implicated in the regulation of calcification in the extra-pulmonary vasculature. Importantly, vascular calcification also seems to be closely interconnected with ECM remodeling and stiffening (201), as SMC mineralization directly correlates with the production of collagen I and fibronectin and elastin degradation, while the latter forms scaffolds for calcium incorporation (201–203). These findings suggest that upstream inflammation may also promote vascular calcification through ECM remodeling.

## Pulmonary arterial endothelial-to-mesenchymal transition and inflammation

While our interrogation of vascular calcification processes has at large focused on SMCs, it is important to keep in mind that ECs are also involved. In various cardiovascular diseases, ECs lose their characteristic morphology and undergo a shift toward a mesenchymal phenotype (204), a process that is termed endothelial-to-mesenchymal transition (EndoMT) and that is notably modulated by inflammation. Specifically, inflammatory cytokines such as IL-1β or TNF have been shown to induce EndoMT in PA ECs. In turn, these EndoMT cells start to secrete inflammatory cytokines including IL-4, IL-6, IL-8, IL-13, and TNF at much higher concentrations as compared to normal PA ECs (205), thus establishing a potentially vicious feed-forward loop. In line with the notion of inflammation-driven EndoMT in PH, activation of the pro-inflammatory NF-κB signaling pathway in a mouse model of monocrotaline (MCT)-induced PH was found to upregulate miR-130a, which induced loss of bone morphogenetic protein receptor type 2 (BMPR2), increased expression of High Mobility Group AT-hook 1 (HMGA1), and ultimately EndoMT in lung microvascular ECs (206). It is important to highlight that although EndoMT has been extensively documented in pulmonary and systemic ECs exposed to inflammatory mediators *in vitro*, the extent and relevance of EndoMT *in vivo* in recent studies using lineage tracing technologies remains controversial: By use of double transgenic mice stably expressing green fluorescent protein (GFP) in all ECs, Suzuki and colleagues detected GFP in 14.3 ± 1.8% of mesenchymal (CD144^-^CD45^-^CD326^-^) cells, indicating substantial EndoMT (207). Similarly, endothelial lineage tracing using transgenic vascular endothelial-cadherin Cre recombinase or Tie-2 Cre mice intercrossed with mTomato/mGreen fluorescent protein double-fluorescent Cre reporter mice revealed abundant endothelial lineage-marked cells in the neointima where they expressed smooth muscle α-actin and smooth muscle myosin heavy chain following induction of PH by monocrotaline pyrrole (208). Yet, a recent lineage tracing study in chronic hypoxia and allergen-induced models of lung vascular remodeling showed retention of endothelial lineage-specific marker expression profiles without any indication of cell-type conversion (209). Notably, the recognition of limited or partial EndoMT does not necessarily conflict with its potential functional relevance in PA stiffening, but simply suggests that this relevance may potentially relate more to the release of proliferative, hypertrophic, and profibrotic signals—i.e., mediators of processes that will ultimately promote PA stiffness—by partial EndoMT cells rather than to the actual generation of significant mesenchymal cell mass *via* this mechanism. Indeed, a similar role is increasingly recognized for epithelial–mesenchymal transition in tissue fibrosis (210).

Over and above that, EndoMT may link inflammation to vascular calcification and, thus, PA stiffening in PH. Specifically, studies in aortic ECs show that inflammatory cytokines such as TNF and IL-1β modulate EndoMT and downregulate the expression of BMPR2 and JNK signaling, thereby sensitizing ECs for BMP9-induced osteogenic differentiation that culminates in mineralization (141). Similar regulatory mechanisms may drive PA EC calcification in different types of PH, and PAH patients with BMPR2 mutations or BMP signaling pathway impairments (104) would be expected to be specifically vulnerable in this scenario given the association of impaired BMPR2 signaling with EndoMT (211). Lineage tracing studies in the systemic circulation support a role for EndoMT in vascular calcification, showing, e.g., that a subset of endocardial cells can undergo endocardial-to-mesenchymal transition resulting in calcification of mouse and human cardiac valves (212) or that vascular ECs can transition into osteogenic cells (213), which can be prevented by inhibition of glycogen synthase kinase 3 (GSK3) (214). The role of EndoMT (or partial EndoMT) in vascular calcification in the pulmonary circulation and in the contact of PH has, however, so far not been addressed.

## Potential clinical relevance

While current PH therapies (i.e., prostacyclins, phosphodiesterase inhibitors, calcium channel blockers, endothelin receptor antagonists, or soluble guanylate cyclase stimulators) focus primarily on alleviating vasoconstriction as a symptomatic approach (215), the long-term therapeutic goal is to shift towards targeting mechanisms of disease onset and progression, including vascular remodeling and inflammation (215). In this regard, targeting the immune–PA stiffening axis may present a particularly promising strategy in light of the predictive and pathomechanistic role of PA stiffening in PH, and the armamentarium of immunomodulatory therapies already in clinical use or in development. In the systemic circulation, anti-inflammatory therapies have shown promise to reduce arterial stiffening in inflammatory artheropathies such as rheumatoid or psoriatic arthritis (167). Specifically, TNF antagonists, such as adalimumab, etanercept, or infliximab, represent established anti-inflammatory therapies in (auto-)immune conditions (216) that have explicitly lowered aortic stiffness in patients with inflammatory artheropathies (107, 167).

As such, immunomodulatory treatments are increasingly considered as potential therapeutic strategies for the treatment of PH. Yet, despite promising findings in preclinical models (8, 146, 215, 217), results from clinical trials have so far shown only modest benefit (149, 218, 219), thus stressing the need for more personalized approaches. Given the discussed link between immune responses, ECM remodeling, and vascular calcification, PA stiffness may present a promising biomarker to identify and monitor patients who may profit from immunomodulatory therapies; yet, assessment of PA stiffness in clinical trials is presently rare. Preclinical models, however, highlight the potential promise of anti-inflammatory therapies to target PA stiffness: For example, inhibition of carbonic anhydrases by acetazolamide or ammonium chloride (NH_4_Cl) as a potential treatment for inflammation in PH was able to prevent SMC dedifferentiation and proliferation in a Sugen5416/hypoxia rat model (220). Other anti-inflammatory therapies, such as treatment with resveratrol, were similarly able to prevent PA remodeling and stiffening in chronic hypoxic rats (215), while inhibitors of the renin–angiotensin system such as captopril or losartan reduced the production of ECM components including interstitial collagen and the expression of MMP-2 and MMP-9 in PAH, thereby attenuating PA stiffening (215). Hence, targeting inflammation with a specific focus on PA stiffness may provide for a pathomechanism-based and individualized therapy to treat PH—a notion that should be considered and, ideally, may be tested in appropriate preclinical and clinical trials.

## Author contributions

S-FL, NNV, and MK conceived and wrote the original draft manuscript. QL drew the figures. MK, CK, and WK conceived and revised the manuscript. All authors contributed to the article and approved the submitted version.

## Funding

WK was supported by grants from the German Research Foundation (CRC 1470, A04; KU1218/12-1) and the German Center for Cardiovascular Research partner site Berlin, CK was supported by the German Centre for Cardiovascular Research partner site Berlin and by the German Ministry of Education and Research (BMBF), NNV was supported by the Doctoral Scholarship from the German Centre for Cardiovascular Research and the German Cardiology Association (DGK), MK was supported by the German Foundation for Heart Research (F23/20), and S-FL and QL were supported by the China Scholarship Council (No. 202008350142 and No.202108080221).

## Acknowledgments

Parts of the figures were generated with the help of BioRender.

## Conflict of interest

The authors declare that the research was conducted in the absence of any commercial or financial relationships that could be construed as a potential conflict of interest.

## Publisher’s note

All claims expressed in this article are solely those of the authors and do not necessarily represent those of their affiliated organizations, or those of the publisher, the editors and the reviewers. Any product that may be evaluated in this article, or claim that may be made by its manufacturer, is not guaranteed or endorsed by the publisher.

## References

[1] Simonneau G Montani D Celermajer DS Denton CP Gatzoulis MA Krowka M. Haemodynamic definitions and updated clinical classification of pulmonary hypertension. Eur Respir J (2019) 53(1):1801913. doi: 10.1183/13993003.01913-2018 30545968 PMC6351336

[2] Condon DF Nickel NP Anderson R Mirza S de Jesus Perez VA. The 6th world symposium on pulmonary hypertension: What's old is new. F1000Res (2019) 8:F1000 Faculty Rev–888. doi: 10.12688/f1000research.18811.1 PMC6584967 31249672

[3] Simonneau G Robbins IM Beghetti M Channick RN Delcroix M Denton CP. Updated clinical classification of pulmonary hypertension. J Am Coll Cardiol (2009) 54:S43–54. doi: 10.1016/j.jacc.2009.04.012 19555858

[4] Galie N Humbert M Vachiery JL Gibbs S Lang I Torbicki A. ESC/ERS guidelines for the diagnosis and treatment of pulmonary hypertension: The joint task force for the diagnosis and treatment of pulmonary hypertension of the European society of cardiology (ESC) and the European respiratory society (ERS): Endorsed by: Association for European paediatric and congenital cardiology (AEPC), international society for heart and lung transplantation (ISHLT). Eur Respir J (2015) 46:903–75. doi: 10.1183/13993003.01032-2015 26318161

[5] Lan NSH Massam BD Kulkarni SS Lang CC. Pulmonary arterial hypertension: Pathophysiology and treatment. Diseases (2018) 6 (2):38. doi: 10.3390/diseases6020038 PMC6023499 29772649

[6] Savai R Pullamsetti SS Kolbe J Bieniek E Voswinckel R Fink L. Immune and inflammatory cell involvement in the pathology of idiopathic pulmonary arterial hypertension. Am J Respir Crit Care Med (2012) 186:897–908. doi: 10.1164/rccm.201202-0335OC 22955318

[7] El Kasmi KC Pugliese SC Riddle SR Poth JM Anderson AL Frid MG. Adventitial fibroblasts induce a distinct proinflammatory/profibrotic macrophage phenotype in pulmonary hypertension. J Immunol (2014) 193:597–609. doi: 10.4049/jimmunol.1303048 24928992 PMC4100597

[8] Breitling S Hui Z Zabini D Hu Y Hoffmann J Goldenberg NM. The mast cell-b cell axis in lung vascular remodeling and pulmonary hypertension. Am J Physiol Lung Cell Mol Physiol (2017) 312:L710–21. doi: 10.1152/ajplung.00311.2016 28235950

[9] Pugliese SC Poth JM Fini MA Olschewski A El Kasmi KC Stenmark KR. The role of inflammation in hypoxic pulmonary hypertension: From cellular mechanisms to clinical phenotypes. Am J Physiol Lung Cell Mol Physiol (2015) 308:L229–252. doi: 10.1152/ajplung.00238.2014 PMC4338929 25416383

[10] Soon E Holmes AM Treacy CM Doughty NJ Southgate L Machado RD. Elevated levels of inflammatory cytokines predict survival in idiopathic and familial pulmonary arterial hypertension. Circulation (2010) 122:920–7. doi: 10.1161/CIRCULATIONAHA.109.933762 20713898

[11] Rabinovitch M Guignabert C Humbert M Nicolls MR. Inflammation and immunity in the pathogenesis of pulmonary arterial hypertension. Circ Res (2014) 115:165–75. doi: 10.1161/CIRCRESAHA.113.301141 PMC4097142 24951765

[12] Spiekerkoetter E Goncharova EA Guignabert C Stenmark K Kwapiszewska G Rabinovitch M. Hot topics in the mechanisms of pulmonary arterial hypertension disease: Cancer-like pathobiology, the role of the adventitia, systemic involvement, and right ventricular failure. Pulm Circ (2019) 9:2045894019889775. doi: 10.1177/2045894019889775 31798835 PMC6868582

[13] Kuebler WM Bonnet S Tabuchi A. Inflammation and autoimmunity in pulmonary hypertension: Is there a role for endothelial adhesion molecules? (2017 grover conference series). Pulm Circ (2018) 8:2045893218757596. doi: 10.1177/2045893218757596 29480134 PMC5865459

[14] Hu Y Chi L Kuebler WM Goldenberg NM. Perivascular inflammation in pulmonary arterial hypertension. Cells (2020) 9 (11):2338. doi: 10.3390/cells9112338 PMC7690279 33105588

[15] Sasaki N Kamataki A Sawai T. A histopathological study of pulmonary hypertension in connective tissue disease. Allergol Int (2011) 60:411–7. doi: 10.2332/allergolint.11-RAI-0337 21918364

[16] Humbert M Monti G Brenot F Sitbon O Portier A Grangeot-Keros L. Increased interleukin-1 and interleukin-6 serum concentrations in severe primary pulmonary hypertension. Am J Respir Crit Care Med (1995) 151:1628–31. doi: 10.1164/ajrccm.151.5.7735624 7735624

[17] Breitling S Ravindran K Goldenberg NM Kuebler WM. The pathophysiology of pulmonary hypertension in left heart disease. Am J Physiol Lung Cell Mol Physiol (2015) 309:L924–941. doi: 10.1152/ajplung.00146.2015 26502478

[18] Gan CT Lankhaar JW Westerhof N Marcus JT Becker A Twisk JW. Noninvasively assessed pulmonary artery stiffness predicts mortality in pulmonary arterial hypertension. Chest (2007) 132:1906–12. doi: 10.1378/chest.07-1246 17989161

[19] Agoston-Coldea L Lupu S Mocan T. Pulmonary artery stiffness by cardiac magnetic resonance imaging predicts major adverse cardiovascular events in patients with chronic obstructive pulmonary disease. Sci Rep (2018) 8:14447. doi: 10.1038/s41598-018-32784-6 30262820 PMC6160404

[20] Al-Naamani N Preston IR Paulus JK Hill NS Roberts KE. Pulmonary arterial capacitance is an important predictor of mortality in heart failure with a preserved ejection fraction. JACC Heart Fail (2015) 3:467–74. doi: 10.1016/j.jchf.2015.01.013 PMC4536851 26046840

[21] Wang Z Chesler NC. Pulmonary vascular wall stiffness: An important contributor to the increased right ventricular afterload with pulmonary hypertension. Pulm Circ (2011) 1:212–23. doi: 10.4103/2045-8932.83453 PMC3198648 22034607

[22] Papolos A Tison GH Mayfield J Vasti E DeMarco T. Echocardiographic assessment of pulmonary arterial capacitance predicts mortality in pulmonary hypertension. J Cardiol (2021) 77:279–84. doi: 10.1016/j.jjcc.2020.10.006 PMC8452137 33158713

[23] Sugimoto K Yoshihisa A Nakazato K Jin Y Suzuki S Yokokawa T. Pulmonary arterial capacitance predicts cardiac events in pulmonary hypertension due to left heart disease. PloS One (2016) 11:e0165603. doi: 10.1371/journal.pone.0165603 27875533 PMC5119730

[24] Friedberg MK Feinstein JA Rosenthal DN. Noninvasive assessment of pulmonary arterial capacitance by echocardiography. J Am Soc Echocardiogr (2007) 20:186–90. doi: 10.1016/j.echo.2006.08.009 17275705

[25] Dieffenbach PB Maracle M Tschumperlin DJ Fredenburgh LE. Mechanobiological feedback in pulmonary vascular disease. Front Physiol (2018) 9:951. doi: 10.3389/fphys.2018.00951 30090065 PMC6068271

[26] Reuben SR. Compliance of the human pulmonary arterial system in disease. Circ Res (1971) 29:40–50. doi: 10.1161/01.RES.29.1.40 5561407

[27] Tedford RJ Hassoun PM Mathai SC Girgis RE Russell SD Thiemann DR. Pulmonary capillary wedge pressure augments right ventricular pulsatile loading. Circulation (2012) 125:289–97. doi: 10.1161/CIRCULATIONAHA.111.051540 PMC3264431 22131357

[28] Lin MT Chen YS Huang SC Chiu HH Chiu SN Chen CA. Alternative approach for selected severe pulmonary hypertension of congenital heart defect without initial correction–palliative surgical treatment. Int J Cardiol (2011) 151:313–7. doi: 10.1016/j.ijcard.2010.05.067 20580107

[29] Wagenvoort CA Wagenvoort N Draulans-Noe Y. Reversibility of plexogenic pulmonary arteriopathy following banding of the pulmonary artery. J Thorac Cardiovasc Surg (1984) 87:876–86. doi: 10.1016/S0022-5223(19)38415-6 6727410

[30] Cho JY Kim KH. Evaluation of arterial stiffness by echocardiography: Methodological aspects. Chonnam Med J (2016) 52:101–6. doi: 10.4068/cmj.2016.52.2.101 PMC4880573 27231673

[31] Cheng XL He JG Liu ZH Gu Q Ni XH Zhao ZH. Pulmonary vascular capacitance is associated with vasoreactivity and long-term response to calcium channel blockers in idiopathic pulmonary arterial hypertension. Lung (2016) 194:613–8. doi: 10.1007/s00408-016-9905-0 27272652

[32] Shafie D Dohaei A Amin A Taghavi S Naderi N. Pulmonary vascular capacitance as a predictor of vasoreactivity in idiopathic pulmonary arterial hypertension tested by adenosine. Res Cardiovasc Med (2015) 4(4):e28945. doi: 10.5812/cardiovascmed.28945 26528452 PMC4623377

[33] Sajan I Manlhiot C Reyes J McCrindle BW Humpl T Friedberg MK. Pulmonary arterial capacitance in children with idiopathic pulmonary arterial hypertension and pulmonary arterial hypertension associated with congenital heart disease: relation to pulmonary vascular resistance, exercise capacity, and survival. Am Heart J (2011) 162:562–8. doi: 10.1016/j.ahj.2011.06.014 21884877

[34] Pellegrini P Rossi A Pasotti M Raineri C Cicoira M Bonapace S. Prognostic relevance of pulmonary arterial compliance in patients with chronic heart failure. Chest (2014) 145:1064–70. doi: 10.1378/chest.13-1510 24356904

[35] Dupont M Mullens W Skouri HN Abrahams Z Wu Y Taylor DO. Prognostic role of pulmonary arterial capacitance in advanced heart failure. Circ Heart Fail (2012) 5:778–85. doi: 10.1161/CIRCHEARTFAILURE.112.968511 PMC3538355 23087402

[36] Dragu R Rispler S Habib M Sholy H Hammerman H Galie N. Pulmonary arterial capacitance in patients with heart failure and reactive pulmonary hypertension. Eur J Heart Fail (2015) 17:74–80. doi: 10.1002/ejhf.192 25388783

[37] Saito Y Ohtani T Kioka H Onishi T Tsukamoto Y Nakamoto K. Clinical significance of pulmonary arterial capacitance calculated by echocardiography in patients with advanced heart failure. Circ J (2017) 81:1871–8. doi: 10.1253/circj.CJ-16-1318 28679970

[38] Sanz J Kariisa M Dellegrottaglie S Prat-Gonzalez S Garcia MJ Fuster V. Evaluation of pulmonary artery stiffness in pulmonary hypertension with cardiac magnetic resonance. JACC Cardiovasc Imaging (2009) 2:286–95. doi: 10.1016/j.jcmg.2008.08.007 19356573

[39] Botney MD Kaiser LR Cooper JD Mecham RP Parghi D Roby J. Extracellular matrix protein gene expression in atherosclerotic hypertensive pulmonary arteries. Am J Pathol (1992) 140:357–64. PMC1886436 1739129

[40] Kohn JC Lampi MC Reinhart-King CA. Age-related vascular stiffening: Causes and consequences. Front Genet (2015) 6:112. doi: 10.3389/fgene.2015.00112 25926844 PMC4396535

[41] Wagenseil JE Mecham RP. Vascular extracellular matrix and arterial mechanics. Physiol Rev (2009) 89:957–89. doi: 10.1152/physrev.00041.2008 PMC2775470 19584318

[42] Wagenseil JE Mecham RP. Elastin in large artery stiffness and hypertension. J Cardiovasc Transl Res (2012) 5:264–73. doi: 10.1007/s12265-012-9349-8 PMC3383658 22290157

[43] Thenappan T Chan SY Weir EK. Role of extracellular matrix in the pathogenesis of pulmonary arterial hypertension. Am J Physiol Heart Circ Physiol (2018) 315:H1322–31. doi: 10.1152/ajpheart.00136.2018 PMC6297810 30141981

[44] Botney MD Liptay MJ Kaiser LR Cooper JD Parks WC Mecham RP. Active collagen synthesis by pulmonary arteries in human primary pulmonary hypertension. Am J Pathol (1993) 143:121–9. PMC1886956 7686340

[45] Hoffmann J Marsh LM Pieper M Stacher E Ghanim B Kovacs G. Compartment-specific expression of collagens and their processing enzymes in intrapulmonary arteries of IPAH patients. Am J Physiol Lung Cell Mol Physiol (2015) 308:L1002–1013. doi: 10.1152/ajplung.00383.2014 PMC4437007 25840998

[46] Stenmark KR Yeager ME El Kasmi KC Nozik-Grayck E Gerasimovskaya EV Li M. The adventitia: Essential regulator of vascular wall structure and function. Annu Rev Physiol (2013) 75:23–47. doi: 10.1146/annurev-physiol-030212-183802 23216413 PMC3762248

[47] Chi PL Cheng CC Hung CC Wang MT Liu HY Ke MW. MMP-10 from M1 macrophages promotes pulmonary vascular remodeling and pulmonary arterial hypertension. Int J Biol Sci (2022) 18:331–48. doi: 10.7150/ijbs.66472 PMC8692144 34975336

[48] Bertero T Oldham WM Cottrill KA Pisano S Vanderpool RR Yu Q. Vascular stiffness mechanoactivates YAP/TAZ-dependent glutaminolysis to drive pulmonary hypertension. J Clin Invest (2016) 126:3313–35. doi: 10.1172/JCI86387 PMC5004943 27548520

[49] McEniery CM Wilkinson IB. Large Artery stiffness and inflammation. J Hum Hypertens (2005) 19:507–9. doi: 10.1038/sj.jhh.1001814 15647777

[50] Hardy E Hardy-Sosa A Fernandez-Patron C. MMP-2: Is too low as bad as too high in the cardiovascular system? Am J Physiol Heart Circ Physiol (2018) 315:H1332–40. doi: 10.1152/ajpheart.00198.2018 30118342

[51] Loffek S Schilling O Franzke CW. Series "matrix metalloproteinases in lung health and disease": Biological role of matrix metalloproteinases: A critical balance. Eur Respir J (2011) 38:191–208. doi: 10.1183/09031936.00146510 21177845

[52] Lepetit H Eddahibi S Fadel E Frisdal E Munaut C Noel A. Smooth muscle cell matrix metalloproteinases in idiopathic pulmonary arterial hypertension. Eur Respir J (2005) 25:834–42. doi: 10.1183/09031936.05.00072504 15863640

[53] Golob MJ Tabima DM Wolf GD Johnston JL Forouzan O Mulchrone AM. Pulmonary arterial strain- and remodeling-induced stiffening are differentiated in a chronic model of pulmonary hypertension. J Biomech (2017) 55:92–8. doi: 10.1016/j.jbiomech.2017.02.003 PMC5535793 28262286

[54] Demer LL Tintut Y. Vascular calcification: Pathobiology of a multifaceted disease. Circulation (2008) 117:2938–48. doi: 10.1161/CIRCULATIONAHA.107.743161 PMC4431628 18519861

[55] Ruffenach G Chabot S Tanguay VF Courboulin A Boucherat O Potus F. Role for runt-related transcription factor 2 in proliferative and calcified vascular lesions in pulmonary arterial hypertension. Am J Respir Crit Care Med (2016) 194:1273–85. doi: 10.1164/rccm.201512-2380OC 27149112

[56] Sun Y Byon CH Yuan K Chen J Mao X Heath JM. Smooth muscle cell-specific runx2 deficiency inhibits vascular calcification. Circ Res (2012) 111:543–52. doi: 10.1161/CIRCRESAHA.112.267237 PMC3678289 22773442

[57] Friesen RM Schafer M Ivy DD Abman SH Stenmark K Browne LP. Proximal pulmonary vascular stiffness as a prognostic factor in children with pulmonary arterial hypertension. Eur Heart J Cardiovasc Imaging (2019) 20:209–17. doi: 10.1093/ehjci/jey069 PMC6343079 29788051

[58] Li M Tan Y Stenmark KR Tan W. High pulsatility flow induces acute endothelial inflammation through overpolarizing cells to activate NF-kappaB. Cardiovasc Eng Technol (2013) 4:26–38. doi: 10.1007/s13239-012-0115-5 23667401 PMC3646301

[59] Steppan J Barodka V Berkowitz DE Nyhan D. Vascular stiffness and increased pulse pressure in the aging cardiovascular system. Cardiol Res Pract (2011) 2011:263585. doi: 10.4061/2011/263585 21845218 PMC3154449

[60] Steiner MK Syrkina OL Kolliputi N Mark EJ Hales CA Waxman AB. Interleukin-6 overexpression induces pulmonary hypertension. Circ Res (2009) 104:236–44. doi: 10.1161/CIRCRESAHA.108.182014 PMC5482545 19074475

[61] Huang W Liu H Pan Y Yang H Lin J Zhang H. Mechanical stretching of the pulmonary vein mediates pulmonary hypertension due to left heart disease by regulating SAC/MAPK pathway and the expression of IL-6 and TNF-alpha. J Cardiothorac Surg (2021) 16:127. doi: 10.1186/s13019-021-01471-5 33971931 PMC8107413

[62] Hurlimann D Forster A Noll G Enseleit F Chenevard R Distler O. Anti-tumor necrosis factor-alpha treatment improves endothelial function in patients with rheumatoid arthritis. Circulation (2002) 106:2184–7. doi: 10.1161/01.CIR.0000037521.71373.44 12390945

[63] Zhu Y Huang Y Ji Q Fu S Gu J Tai N. Interplay between extracellular matrix and neutrophils in diseases. J Immunol Res (2021) 2021:8243378. doi: 10.1155/2021/8243378 34327245 PMC8302397

[64] Tenkorang MAA Chalise U Daseke Ii MJ Konfrst SR Lindsey ML. Understanding the mechanisms that determine extracellular matrix remodeling in the infarcted myocardium. Biochem Soc Trans (2019) 47:1679–87. doi: 10.1042/BST20190113 31724697

[65] Ma Z Mao C Jia Y Fu Y Kong W. Extracellular matrix dynamics in vascular remodeling. Am J Physiol Cell Physiol (2020) 319:C481–99. doi: 10.1152/ajpcell.00147.2020 PMC7509265 32579472

[66] Udjus C Cero FT Halvorsen B Behmen D Carlson CR Bendiksen BA. Caspase-1 induces smooth muscle cell growth in hypoxia-induced pulmonary hypertension. Am J Physiol Lung Cell Mol Physiol (2019) 316:L999–L1012. doi: 10.1152/ajplung.00322.2018 30908936

[67] Parpaleix A Amsellem V Houssaini A Abid S Breau M Marcos E. Role of interleukin-1 receptor 1/MyD88 signalling in the development and progression of pulmonary hypertension. Eur Respir J (2016) 48:470–83. doi: 10.1183/13993003.01448-2015 27418552

[68] Tang C Luo Y Li S Huang B Xu S Li L. Characteristics of inflammation process in monocrotaline-induced pulmonary arterial hypertension in rats. BioMed Pharmacother (2021) 133:111081. doi: 10.1016/j.biopha.2020.111081 33378977

[69] Koudstaal T van Hulst JAC Das T Neys SFH Merkus D Bergen IM. DNGR1-cre-mediated deletion of Tnfaip3/A20 in conventional dendritic cells induces pulmonary hypertension in mice. Am J Respir Cell Mol Biol (2020) 63:665–80. doi: 10.1165/rcmb.2019-0443OC 32755457

[70] Wang J Tian XT Peng Z Li WQ Cao YY Li Y. HMGB1/TLR4 promotes hypoxic pulmonary hypertension *via* suppressing BMPR2 signaling. Vascul Pharmacol (2019) 117:35–44. doi: 10.1016/j.vph.2018.12.006 30610955

[71] Li M Riddle SR Frid MG El Kasmi KC McKinsey TA Sokol RJ. Emergence of fibroblasts with a proinflammatory epigenetically altered phenotype in severe hypoxic pulmonary hypertension. J Immunol (2011) 187:2711–22. doi: 10.4049/jimmunol.1100479 PMC3159707 21813768

[72] Smolders V Lodder K Rodriguez C Tura-Ceide O Barbera JA Jukema JW. The inflammatory profile of CTEPH-derived endothelial cells is a possible driver of disease progression. Cells (2021) 10(4):737. doi: 10.3390/cells10040737 PMC8067175 33810533

[73] Gaillard V Casellas D Seguin-Devaux C Schohn H Dauca M Atkinson J. Pioglitazone improves aortic wall elasticity in a rat model of elastocalcinotic arteriosclerosis. Hypertension (2005) 46:372–9. doi: 10.1161/01.HYP.0000171472.24422.33 15967870

[74] Santiago JJ Dangerfield AL Rattan SG Bathe KL Cunnington RH Raizman JE. Cardiac fibroblast to myofibroblast differentiation *in vivo* and *in vitro*: expression of focal adhesion components in neonatal and adult rat ventricular myofibroblasts. Dev Dyn (2010) 239:1573–84. doi: 10.1002/dvdy.22280 20503355

[75] Sanchez-Duffhues G Garcia de Vinuesa A van de Pol V Geerts ME de Vries MR Janson SG. Inflammation induces endothelial-to-mesenchymal transition and promotes vascular calcification through downregulation of BMPR2. J Pathol (2019) 247:333–46. doi: 10.1002/path.5193 PMC6590480 30430573

[76] Tomaszewski M Grywalska E Tomaszewski A Blaszczak P Kurzyna M Rolinski J. Overexpression of PD-1 on peripheral blood lymphocytes in patients with idiopathic pulmonary arterial hypertension and its association with high viral loads of Epstein-Barr virus and poor clinical parameters. J Clin Med (2020) 9(6):1966. doi: 10.3390/jcm9061966 PMC7355537 32599687

[77] Majeed B Tawinwung S Eberson LS Secomb TW Larmonier N Larson DF. Interleukin-2/Anti-Interleukin-2 immune complex expands regulatory T cells and reduces angiotensin II-induced aortic stiffening. Int J Hypertens (2014) 2014:126365. doi: 10.1155/2014/126365 25258681 PMC4167213

[78] Chaouat A Savale L Chouaid C Tu L Sztrymf B Canuet M. Role for interleukin-6 in COPD-related pulmonary hypertension. Chest (2009) 136:678–87. doi: 10.1378/chest.08-2420 19349390

[79] Sweatt AJ Hedlin HK Balasubramanian V Hsi A Blum LK Robinson WH. Discovery of distinct immune phenotypes using machine learning in pulmonary arterial hypertension. Circ Res (2019) 124:904–19. doi: 10.1161/CIRCRESAHA.118.313911 PMC6428071 30661465

[80] Koudstaal T van Uden D van Hulst JAC Heukels P Bergen IM Geenen LW. Plasma markers in pulmonary hypertension subgroups correlate with patient survival. Respir Res (2021) 22:137. doi: 10.1186/s12931-021-01716-w 33947407 PMC8097895

[81] Ranchoux B Nadeau V Bourgeois A Provencher S Tremblay E Omura J. Metabolic syndrome exacerbates pulmonary hypertension due to left heart disease. Circ Res (2019) 125:449–66. doi: 10.1161/CIRCRESAHA.118.314555 31154939

[82] Hashimoto-Kataoka T Hosen N Sonobe T Arita Y Yasui T Masaki T. Interleukin-6/interleukin-21 signaling axis is critical in the pathogenesis of pulmonary arterial hypertension. Proc Natl Acad Sci U.S.A. (2015) 112:E2677–2686. doi: 10.1073/pnas.1424774112 PMC4443312 25941359

[83] Batah SS Alda MA Rodrigues Lopes Roslindo Figueira R Cruvinel HR Perdona Rodrigues da Silva L Machado-Rugolo J. *In situ* evidence of collagen V and interleukin-6/Interleukin-17 activation in vascular remodeling of experimental pulmonary hypertension. Pathobiol (2020) 87:356–66. doi: 10.1159/000510048 33099553

[84] Simpson CE Chen JY Damico RL Hassoun PM Martin LJ Yang J. Cellular sources of interleukin-6 and associations with clinical phenotypes and outcomes in pulmonary arterial hypertension. Eur Respir J (2020) 55(4):1901761. doi: 10.1183/13993003.01761-2019 PMC8243361 32029443

[85] He H Tao Y Chen X Qiu H Zhu J Zhang J. High levels of interleukin-6 and 8-iso-prostaglandin in the exhaled breath condensate and serum of patients with chronic obstructive pulmonary disease related pulmonary hypertension. Chin Med J (Engl) (2014) 127:1608–12. 24791862

[86] Khan YM Kirkham P Barnes PJ Adcock IM. Brd4 is essential for IL-1beta-induced inflammation in human airway epithelial cells. PloS One (2014) 9(4):e95051. doi: 10.1371/journal.pone.0095051 24759736 PMC3997389

[87] Meloche J Potus F Vaillancourt M Bourgeois A Johnson I Deschamps L. Bromodomain-containing protein 4: The epigenetic origin of pulmonary arterial hypertension. Circ Res (2015) 117:525–35. doi: 10.1161/CIRCRESAHA.115.307004 26224795

[88] Maniatis K Siasos G Oikonomou E Vavuranakis M Zaromytidou M Mourouzis K. Osteoprotegerin and osteopontin serum levels are associated with vascular function and inflammation in coronary artery disease patients. Curr Vasc Pharmacol (2020) 18:523–30. doi: 10.2174/1570161117666191022095246 31642412

[89] Gokaslan S Ozer Gokaslan C Demirel E Celik S. Role of aortic stiffness and inflammation in the etiology of young-onset hypertension. Turk J Med Sci (2019) 49(6):1748–53. doi: 10.3906/sag-1908-137 PMC7518685 31655529

[90] Peyster E Chen J Feldman HI Go AS Gupta J Mitra N. Inflammation and arterial stiffness in chronic kidney disease: Findings from the CRIC study. Am J Hypertens (2017) 30:400–8. doi: 10.1093/ajh/hpw164 PMC5861572 28391349

[91] Quarck R Wynants M Verbeken E Meyns B Delcroix M. Contribution of inflammation and impaired angiogenesis to the pathobiology of chronic thromboembolic pulmonary hypertension. Eur Respir J (2015) 46:431–43. doi: 10.1183/09031936.00009914 26113681

[92] Sikka G Miller KL Steppan J Pandey D Jung SM Fraser CD 3rd. Interleukin 10 knockout frail mice develop cardiac and vascular dysfunction with increased age. . Exp Gerontol (2013) 48:128–35. doi: 10.1016/j.exger.2012.11.001 PMC3744178 23159957

[93] Calvier L Legchenko E Grimm L Sallmon H Hatch A Plouffe BD. Galectin-3 and aldosterone as potential tandem biomarkers in pulmonary arterial hypertension. Heart (2016) 102:390–6. doi: 10.1136/heartjnl-2015-308365 26869635

[94] Larsen KO Yndestad A Sjaastad I Loberg EM Goverud IL Halvorsen B. Lack of CCR7 induces pulmonary hypertension involving perivascular leukocyte infiltration and inflammation. Am J Physiol Lung Cell Mol Physiol (2011) 301:L50–59. doi: 10.1152/ajplung.00048.2010 21498626

[95] Wang W Yan H Zhu W Cui Y Chen J Wang X. Impairment of monocyte-derived dendritic cells in idiopathic pulmonary arterial hypertension. J Clin Immunol (2009) 29:705–13. doi: 10.1007/s10875-009-9322-8 19693657

[96] Yong K Dogra G Boudville N Chan D Adams L Ching H. Interleukin-12 is associated with arterial stiffness in healthy individuals. Am J Hypertens (2013) 26:159–62. doi: 10.1093/ajh/hps032 23382399

[97] Zhu R Xie X Wang N Chen L Hong Y. The T helper type 17/regulatory T cell imbalance was associated with ras-GTPase overexpression in patients with pulmonary hypertension associated with chronic obstructive pulmonary disease. Immunology (2019) 157(4):304–11. doi: 10.1111/imm.13084 PMC6620191 31141166

[98] Huang LH Zinselmeyer BH Chang CH Saunders BT Elvington A Baba O. Interleukin-17 drives interstitial entrapment of tissue lipoproteins in experimental psoriasis. Cell Metab (2019) 29:475–487 e477. doi: 10.1016/j.cmet.2018.10.006 30415924 PMC6365189

[99] Yang M Deng C Wu D Zhong Z Lv X Huang Z. The role of mononuclear cell tissue factor and inflammatory cytokines in patients with chronic thromboembolic pulmonary hypertension. J Thromb Thrombol (2016) 42:38–45. doi: 10.1007/s11239-015-1323-2 PMC4877417 26667361

[100] Saleby J Bouzina H Lundgren J Radegran G. Angiogenic and inflammatory biomarkers in the differentiation of pulmonary hypertension. Scand Cardiovasc J (2017) 51:261–70. doi: 10.1080/14017431.2017.1359419 28776404

[101] Kylhammar D Hesselstrand R Nielsen S Scheele C Radegran G. Angiogenic and inflammatory biomarkers for screening and follow-up in patients with pulmonary arterial hypertension. Scand J Rheumatol (2018) 47:319–24. doi: 10.1080/03009742.2017.1378714 29528272

[102] Naito A Sakao S Terada J Iwasawa S Jujo Sanada T Suda R. Nocturnal hypoxemia and high circulating TNF-alpha levels in chronic thromboembolic pulmonary hypertension. Intern Med (2020) 59:1819–26. doi: 10.2169/internalmedicine.4458-20 PMC7475001 32741891

[103] Bai P Lyu L Yu T Zuo C Fu J He Y. Macrophage-derived legumain promotes pulmonary hypertension by activating the MMP (Matrix metalloproteinase)-2/TGF (Transforming growth factor)-beta1 signaling. Arterioscler Thromb Vasc Biol (2019) 39(4):e130–45. doi: 10.1161/ATVBAHA.118.312254 30676070

[104] Tintut Y Patel J Parhami F Demer LL. Tumor necrosis factor-alpha promotes *in vitro* calcification of vascular cells *via* the cAMP pathway. Circulation (2000) 102:2636–42. doi: 10.1161/01.CIR.102.21.2636 11085968

[105] Pina T Corrales A Lopez-Mejias R Armesto S Gonzalez-Lopez MA Gomez-Acebo I. Anti-tumor necrosis factor-alpha therapy improves endothelial function and arterial stiffness in patients with moderate to severe psoriasis: A 6-month prospective study. J Dermatol (2016) 43:1267–72. doi: 10.1111/1346-8138.13398 27062420

[106] Moreau KL Deane KD Meditz AL Kohrt WM. Tumor necrosis factor-alpha inhibition improves endothelial function and decreases arterial stiffness in estrogen-deficient postmenopausal women. Atherosclerosis (2013) 230:390–6. doi: 10.1016/j.atherosclerosis.2013.07.057 PMC3815590 24075772

[107] Angel K Provan SA Gulseth HL Mowinckel P Kvien TK Atar D. Tumor necrosis factor-alpha antagonists improve aortic stiffness in patients with inflammatory arthropathies: a controlled study. Hypertension (2010) 55:333–8. doi: 10.1161/HYPERTENSIONAHA.109.143982 20038753

[108] Kumar R Mickael C Chabon J Gebreab L Rutebemberwa A Garcia AR. The causal role of IL-4 and IL-13 in schistosoma mansoni pulmonary hypertension. Am J Respir Crit Care Med (2015) 192:998–1008. doi: 10.1164/rccm.201410-1820OC 26192556 PMC4642207

[109] Li S Ma X Xie J Yan X Sun W. MicroRNA-206, IL-4, IL-13, and INF-gamma levels in lung tissue and plasma are increased by the stimulation of particulate matter with a diameter of </=2.5mum, and are associated with the poor prognosis of asthma induced pulmonary arterial hypertension patients. Clin Exp Hypertens (2021) 43:181–8. doi: 10.1080/10641963.2020.1836192 33086901

[110] Cero FT Hillestad V Sjaastad I Yndestad A Aukrust P Ranheim T. Absence of the inflammasome adaptor ASC reduces hypoxia-induced pulmonary hypertension in mice. Am J Physiol Lung Cell Mol Physiol (2015) 309:L378–387. doi: 10.1152/ajplung.00342.2014 26071556

[111] Shao D Perros F Caramori G Meng C Dormuller P Chou PC. Nuclear IL-33 regulates soluble ST2 receptor and IL-6 expression in primary human arterial endothelial cells and is decreased in idiopathic pulmonary arterial hypertension. Biochem Biophys Res Commun (2014) 451:8–14. doi: 10.1016/j.bbrc.2014.06.111 25003325

[112] Carlomagno G Messalli G Melillo RM Stanziola AA Visciano C Mercurio V. Serum soluble ST2 and interleukin-33 levels in patients with pulmonary arterial hypertension. Int J Cardiol (2013) 168:1545–7. doi: 10.1016/j.ijcard.2012.12.031 23290950

[113] Liu J Wang W Wang L Chen S Tian B Huang K. IL-33 initiates vascular remodelling in hypoxic pulmonary hypertension by up-regulating HIF-1alpha and VEGF expression in vascular endothelial cells. EBioMedicine (2018) 33:196–210. doi: 10.1016/j.ebiom.2018.06.003 29921553 PMC6085568

[114] Amsellem V Abid S Poupel L Parpaleix A Rodero M Gary-Bobo G. Roles for the CX3CL1/CX3CR1 and CCL2/CCR2 chemokine systems in hypoxic pulmonary hypertension. Am J Respir Cell Mol Biol (2017) 56:597–608. doi: 10.1165/rcmb.2016-0201OC 28125278

[115] Abid S Marcos E Parpaleix A Amsellem V Breau M Houssaini A. CCR2/CCR5-mediated macrophage-smooth muscle cell crosstalk in pulmonary hypertension. Eur Respir J (2019) 54(4):1802308. doi: 10.1183/13993003.02308-2018 31320454

[116] Bakouboula B Morel O Faure A Zobairi F Jesel L Trinh A. Procoagulant membrane microparticles correlate with the severity of pulmonary arterial hypertension. Am J Respir Crit Care Med (2008) 177:536–43. doi: 10.1164/rccm.200706-840OC 18006886

[117] Chang FC Chiang WC Tsai MH Chou YH Pan SY Chang YT. Angiopoietin-2-induced arterial stiffness in CKD. J Am Soc Nephrol (2014) 25:1198–209. doi: 10.1681/ASN.2013050542 PMC4033368 24511140

[118] Qi D Wei M Jiao S Song Y Wang X Xie G. Hypoxia inducible factor 1alpha in vascular smooth muscle cells promotes angiotensin II-induced vascular remodeling *via* activation of CCL7-mediated macrophage recruitment. Cell Death Dis (2019) 10:544. doi: 10.1038/s41419-019-1757-0 31320613 PMC6639417

[119] Nie X Tan J Dai Y Liu Y Zou J Sun J. CCL5 deficiency rescues pulmonary vascular dysfunction, and reverses pulmonary hypertension *via* caveolin-1-dependent BMPR2 activation. J Mol Cell Cardiol (2018) 116:41–56. doi: 10.1016/j.yjmcc.2018.01.016 29374556

[120] Amsellem V Lipskaia L Abid S Poupel L Houssaini A Quarck R. CCR5 as a treatment target in pulmonary arterial hypertension. Circulation (2014) 130:880–91. doi: 10.1161/CIRCULATIONAHA.114.010757 PMC4160408 24993099

[121] Zeng Y Li N Zheng Z Chen R Peng M Liu W. Screening of hub genes associated with pulmonary arterial hypertension by integrated bioinformatic analysis. BioMed Res Int (2021) 2021:6626094. doi: 10.1155/2021/6626094 33816621 PMC8010527

[122] Dorfmuller P Zarka V Durand-Gasselin I Monti G Balabanian K Garcia G. Chemokine RANTES in severe pulmonary arterial hypertension. Am J Respir Crit Care Med (2002) 165:534–9. doi: 10.1164/ajrccm.165.4.2012112 11850348

[123] Xu J Li X Zhou S Wang R Wu M Tan C. Inhibition of CXCR4 ameliorates hypoxia-induced pulmonary arterial hypertension in rats. Am J Transl Res (2021) 13:1458–70. PMC8014346 33841670

[124] Bordenave J Thuillet R Tu L Phan C Cumont A Marsol C. Neutralization of CXCL12 attenuates established pulmonary hypertension in rats. Cardiovasc Res (2020) 116:686–97. doi: 10.1093/cvr/cvz153 31173066

[125] Olsson KM Olle S Fuge J Welte T Hoeper MM Lerch C. CXCL13 in idiopathic pulmonary arterial hypertension and chronic thromboembolic pulmonary hypertension. Respir Res (2016) 17:21. doi: 10.1186/s12931-016-0336-5 26927848 PMC4770535

[126] Zhang Y Lin P Hong C Jiang Q Xing Y Tang X. Serum cytokine profiles in patients with chronic obstructive pulmonary disease associated pulmonary hypertension identified using protein array. Cytokine (2018) 111:342–9. doi: 10.1016/j.cyto.2018.09.005 30273784

[127] Delaney C Davizon-Castillo P Allawzi A Posey J Gandjeva A Neeves K. Platelet activation contributes to hypoxia-induced inflammation. Am J Physiol Lung Cell Mol Physiol (2021) 320:L413–21. doi: 10.1152/ajplung.00519.2020 PMC8294621 33264579

[128] Zawia A Arnold ND West L Pickworth JA Turton H Iremonger J. Altered macrophage polarization induces experimental pulmonary hypertension and is observed in patients with pulmonary arterial hypertension. Arterioscler Thromb Vasc Biol (2021) 41(1):430–45. doi: 10.1161/ATVBAHA.120.314639 PMC7752239 33147993

[129] Wang L Zhang X Cao Y Ma Q Mao X Xu J. Mice with a specific deficiency of Pfkfb3 in myeloid cells are protected from hypoxia-induced pulmonary hypertension. Br J Pharmacol (2021) 178:1055–72. doi: 10.1111/bph.15339 33300142

[130] Xi X Zhang J Wang J Chen Y Zhang W Zhang X. SGK1 mediates hypoxic pulmonary hypertension through promoting macrophage infiltration and activation. Anal Cell Pathol (Amst) (2019) 2019:3013765. doi: 10.1155/2019/3013765 31815093 PMC6877960

[131] Bochenek ML Rosinus NS Lankeit M Hobohm L Bremmer F Schutz E. From thrombosis to fibrosis in chronic thromboembolic pulmonary hypertension. Thromb Haemost (2017) 117:769–83. doi: 10.1160/TH16-10-0790 28150849

[132] Miao R Dong X Gong J Li Y Guo X Wang J. Cell landscape atlas for patients with chronic thromboembolic pulmonary hypertension after pulmonary endarterectomy constructed using single-cell RNA sequencing. Aging (Albany NY) (2021) 13:16485–99. doi: 10.18632/aging.203168 PMC8266372 34153003

[133] Price LC Caramori G Perros F Meng C Gambaryan N Dorfmuller P. Nuclear factor kappa-b is activated in the pulmonary vessels of patients with end-stage idiopathic pulmonary arterial hypertension. PloS One (2013) 8(10):e75415. doi: 10.1371/journal.pone.0075415 24124488 PMC3790752

[134] Masaki T Okazawa M Asano R Inagaki T Ishibashi T Yamagishi A. Aryl hydrocarbon receptor is essential for the pathogenesis of pulmonary arterial hypertension. Proc Natl Acad Sci U.S.A. (2021) 118(11):e2023899118. doi: 10.1073/pnas.2023899118 33836606 PMC7980441

[135] Lariviere R Gauthier-Bastien A Ung RV St-Hilaire J Mac-Way F Richard DE. Endothelin type a receptor blockade reduces vascular calcification and inflammation in rats with chronic kidney disease. J Hypertens (2017) 35:376–84. doi: 10.1097/HJH.0000000000001161 28005706

[136] Chen JY Wu YP Li CY Jheng HF Kao LZ Yang CC. PPARgamma activation improves the microenvironment of perivascular adipose tissue and attenuates aortic stiffening in obesity. J BioMed Sci (2021) 28:22. doi: 10.1186/s12929-021-00720-y 33781257 PMC8008548

[137] Sharma N Dev R Belenchia AM Aroor AR Whaley-Connell A Pulakat L. Deficiency of IL12p40 (Interleukin 12 p40) promotes ang II (Angiotensin II)-induced abdominal aortic aneurysm. Arterioscler Thromb Vasc Biol (2019) 39:212–23. doi: 10.1161/ATVBAHA.118.311969 PMC6355331 30580570

[138] Tamosiuniene R Manouvakhova O Mesange P Saito T Qian J Sanyal M. Dominant role for regulatory T cells in protecting females against pulmonary hypertension. Circ Res (2018) 122:1689–702. doi: 10.1161/CIRCRESAHA.117.312058 PMC5993601 29545367

[139] Chu Y Xiangli X Xiao W. Regulatory T cells protect against hypoxia-induced pulmonary arterial hypertension in mice. Mol Med Rep (2015) 11:3181–7. doi: 10.3892/mmr.2014.3106 25523119

[140] Ormiston ML Chang C Long LL Soon E Jones D Machado R. Impaired natural killer cell phenotype and function in idiopathic and heritable pulmonary arterial hypertension. Circulation (2012) 126:1099–109. doi: 10.1161/CIRCULATIONAHA.112.110619 22832786

[141] Mao M Zhang M Ge A Ge X Gu R Zhang C. Granzyme b deficiency promotes osteoblastic differentiation and calcification of vascular smooth muscle cells in hypoxic pulmonary hypertension. Cell Death Dis (2018) 9:221. doi: 10.1038/s41419-018-0315-5 29445095 PMC5833422

[142] Maston LD Jones DT Giermakowska W Howard TA Cannon JL Wang W. Central role of T helper 17 cells in chronic hypoxia-induced pulmonary hypertension. Am J Physiol Lung Cell Mol Physiol (2017) 312:L609–24. doi: 10.1152/ajplung.00531.2016 PMC5451600 28213473

[143] Kelly C Mwandumba HC Heyderman RS Jambo K Kamng'ona R Chammudzi M. HIV-Related arterial stiffness in Malawian adults is associated with the proportion of PD-1-Expressing CD8+ T cells and reverses with antiretroviral therapy. J Infect Dis (2019) 219:1948–58. doi: 10.1093/infdis/jiz015 PMC6534190 30629187

[144] Florentin J Zhao J Tai YY Vasamsetti SB O'Neil SP Kumar R. Interleukin-6 mediates neutrophil mobilization from bone marrow in pulmonary hypertension. Cell Mol Immunol (2021) 18:374–84. doi: 10.1038/s41423-020-00608-1 PMC8027442 33420357

[145] Stroka KM Levitan I Aranda-Espinoza H. OxLDL and substrate stiffness promote neutrophil transmigration by enhanced endothelial cell contractility and ICAM-1. J Biomech (2012) 45:1828–34. doi: 10.1016/j.jbiomech.2012.04.011 PMC3376185 22560286

[146] Hoffmann J Yin J Kukucka M Yin N Saarikko I Sterner-Kock A. Mast cells promote lung vascular remodelling in pulmonary hypertension. Eur Respir J (2011) 37:1400–10. doi: 10.1183/09031936.00043310 21148228

[147] Bartelds B van Loon RLE Mohaupt S Wijnberg H Dickinson MG Boersma B. Mast cell inhibition improves pulmonary vascular remodeling in pulmonary hypertension. Chest (2012) 141:651–60. doi: 10.1378/chest.11-0663 21940767

[148] Dahal BK Kosanovic D Kaulen C Cornitescu T Savai R Hoffmann J. Involvement of mast cells in monocrotaline-induced pulmonary hypertension in rats. Respir Res (2011) 12:60. doi: 10.1186/1465-9921-12-60 21535881 PMC3104382

[149] Farha S Sharp J Asosingh K Park M Comhair SA Tang WH. Mast cell number, phenotype, and function in human pulmonary arterial hypertension. Pulm Circ (2012) 2:220–8. doi: 10.4103/2045-8932.97609 PMC3401876 22837863

[150] Weng M Baron DM Bloch KD Luster AD Lee JJ Medoff BD. Eosinophils are necessary for pulmonary arterial remodeling in a mouse model of eosinophilic inflammation-induced pulmonary hypertension. Am J Physiol Lung Cell Mol Physiol (2011) 301:L927–936. doi: 10.1152/ajplung.00049.2011 PMC3233831 21908591

[151] Sun L Ning C Liu J Yao T Zhang L Zhao L. The association between cumulative c-reactive protein and brachial-ankle pulse wave velocity. Aging Clin Exp Res (2020) 32:789–96. doi: 10.1007/s40520-019-01274-8 31352587

[152] Scott HA Quach B Yang X Ardekani S Cabrera AP Wilson R. Matrix stiffness exerts biphasic control over monocyte-endothelial adhesion *via* rho-mediated ICAM-1 clustering. Integr Biol (Camb) (2016) 8:869–78. doi: 10.1039/C6IB00084C 27444067

[153] Deng H Song Z Xu H Deng X Zhang Q Chen H. MicroRNA-1185 promotes arterial stiffness though modulating VCAM-1 and e-selectin expression. Cell Physiol Biochem (2017) 41:2183–93. doi: 10.1159/000475576 28441665

[154] Sun W Chan SY. Pulmonary arterial stiffness: An early and pervasive driver of pulmonary arterial hypertension. Front Med (Lausanne) (2018) 5:204. doi: 10.3389/fmed.2018.00204 30073166 PMC6058030

[155] Wang Z Lakes RS Eickhoff JC Chesler NC. Effects of collagen deposition on passive and active mechanical properties of large pulmonary arteries in hypoxic pulmonary hypertension. Biomech Model Mechanobiol (2013) 12:1115–25. doi: 10.1007/s10237-012-0467-7 PMC3745811 23377784

[156] Franz M Grun K Betge S Rohm I Ndongson-Dongmo B Bauer R. Lung tissue remodelling in MCT-induced pulmonary hypertension: A proposal for a novel scoring system and changes in extracellular matrix and fibrosis associated gene expression. Oncotarget (2016) 7:81241–54. doi: 10.18632/oncotarget.13220 PMC5348389 27835899

[157] Thenappan T Prins KW Pritzker MR Scandurra J Volmers K Weir EK. The critical role of pulmonary arterial compliance in pulmonary hypertension. Ann Am Thorac Soc (2016) 13:276–84. doi: 10.1513/AnnalsATS.201509-599FR PMC5461956 26848601

[158] Moreno J Escobedo D Calhoun C Le Saux CJ Han HC. Arterial wall stiffening in caveolin-1 deficiency-induced pulmonary artery hypertension in mice. Exp Mech (2021) 6:217–28. doi: 10.1007/s11340-020-00666-6 PMC7993546 33776068

[159] Mierke J Christoph M Augstein A Pfluecke C Jellinghaus S Woitek F. Influence of caveolin-1 and endothelial nitric oxide synthase on adventitial inflammation in aortic transplants. Kardiol Pol (2020) 78:124–30. doi: 10.33963/KP.15079 31790082

[160] Bloodworth NC Clark CR West JD Snider JC Gaskill C Shay S. Bone marrow-derived proangiogenic cells mediate pulmonary arteriole stiffening *via* serotonin 2B receptor dependent mechanism. Circ Res (2018) 123:e51–64. doi: 10.1161/CIRCRESAHA.118.313397 PMC6309812 30566041

[161] Quintero-Villegas A Valdes-Ferrer SI. Role of 5-HT7 receptors in the immune system in health and disease. Mol Med (2019) 26:2. doi: 10.1186/s10020-019-0126-x 31892309 PMC6938607

[162] Schafer M Ivy DD Nguyen K Boncella K Frank BS Morgan GJ. Metalloproteinases and their inhibitors are associated with pulmonary arterial stiffness and ventricular function in pediatric pulmonary hypertension. Am J Physiol Heart Circ Physiol (2021) 321:H242–52. doi: 10.1152/ajpheart.00750.2020 PMC8424580 34085841

[163] Lammers S Scott D Hunter K Tan W Shandas R Stenmark KR. Mechanics and function of the pulmonary vasculature: implications for pulmonary vascular disease and right ventricular function. Compr Physiol (2012) 2:295–319. doi: 10.1002/cphy.c100070 PMC3593606 23728987

[164] Protogerou AD Zampeli E Fragiadaki K Stamatelopoulos K Papamichael C Sfikakis PP. A pilot study of endothelial dysfunction and aortic stiffness after interleukin-6 receptor inhibition in rheumatoid arthritis. Atherosclerosis (2011) 219:734–6. doi: 10.1016/j.atherosclerosis.2011.09.015 21968316

[165] Tuttolomondo A Pecoraro R Di Raimondo D Di Sciacca R Canino B Arnao V. Immune-inflammatory markers and arterial stiffness indexes in subjects with acute ischemic stroke with and without metabolic syndrome. Diabetol Metab Syndr (2014) 6:28. doi: 10.1186/1758-5996-6-28 24571954 PMC3942622

[166] Hurst LA Dunmore BJ Long L Crosby A Al-Lamki R Deighton J. TNFalpha drives pulmonary arterial hypertension by suppressing the BMP type-II receptor and altering NOTCH signalling. Nat Commun (2017) 8:14079. doi: 10.1038/ncomms14079 28084316 PMC5241886

[167] Vlachopoulos C Gravos A Georgiopoulos G Terentes-Printzios D Ioakeimidis N Vassilopoulos D. The effect of TNF-a antagonists on aortic stiffness and wave reflections: a meta-analysis. Clin Rheumatol (2018) 37:515–26. doi: 10.1007/s10067-017-3657-y 28484887

[168] Frangogiannis NG. The extracellular matrix in ischemic and nonischemic heart failure. Circ Res (2019) 125:117–46. doi: 10.1161/CIRCRESAHA.119.311148 PMC6588179 31219741

[169] Zhang J Tang L Dai F Qi Y Yang L Liu Z. ROCK inhibitors alleviate myofibroblast transdifferentiation and vascular remodeling *via* decreasing TGFbeta1-mediated RhoGDI expression. Gen Physiol Biophys (2019) 38:271–80. doi: 10.4149/gpb_2019017 31219429

[170] Baum J Duffy HS. Fibroblasts and myofibroblasts: what are we talking about? J Cardiovasc Pharmacol (2011) 57:376–9. doi: 10.1097/FJC.0b013e3182116e39 PMC3077448 21297493

[171] Anwar A Li M Frid MG Kumar B Gerasimovskaya EV Riddle SR. Osteopontin is an endogenous modulator of the constitutively activated phenotype of pulmonary adventitial fibroblasts in hypoxic pulmonary hypertension. Am J Physiol Lung Cell Mol Physiol (2012) 303:L1–L11. doi: 10.1152/ajplung.00050.2012 22582113 PMC3426432

[172] Barman SA Chen F Su Y Dimitropoulou C Wang Y Catravas JD. NADPH oxidase 4 is expressed in pulmonary artery adventitia and contributes to hypertensive vascular remodeling. Arterioscler Thromb Vasc Biol (2014) 34:1704–15. doi: 10.1161/ATVBAHA.114.303848 PMC4228789 24947524

[173] Chazova I Loyd JE Zhdanov VS Newman JH Belenkov Y Meyrick B. Pulmonary artery adventitial changes and venous involvement in primary pulmonary hypertension. Am J Pathol (1995) 146:389–97. PMC1869854 7856750

[174] Chan EC Peshavariya HM Liu GS Jiang F Lim SY Dusting GJ. Nox4 modulates collagen production stimulated by transforming growth factor beta1 *in vivo* and *in vitro* . Biochem Biophys Res Commun (2013) 430:918–25. doi: 10.1016/j.bbrc.2012.11.138 23261430

[175] Lin YC Sung YK Jiang X Peters-Golden M Nicolls MR. Simultaneously targeting myofibroblast contractility and extracellular matrix cross-linking as a therapeutic concept in airway fibrosis. Am J Transplant (2017) 17:1229–41. doi: 10.1111/ajt.14103 PMC5409855 27804215

[176] Nave AH Mizikova I Niess G Steenbock H Reichenberger F Talavera ML. Lysyl oxidases play a causal role in vascular remodeling in clinical and experimental pulmonary arterial hypertension. Arterioscler Thromb Vasc Biol (2014) 34:1446–58. doi: 10.1161/ATVBAHA.114.303534 24833797

[177] Raaz U Schellinger IN Chernogubova E Warnecke C Kayama Y Penov K. Transcription factor Runx2 promotes aortic fibrosis and stiffness in type 2 diabetes mellitus. Circ Res (2015) 117:513–24. doi: 10.1161/CIRCRESAHA.115.306341 PMC4553105 26208651

[178] Bendeck MP Irvin C Reidy MA. Inhibition of matrix metalloproteinase activity inhibits smooth muscle cell migration but not neointimal thickening after arterial injury. Circ Res (1996) 78:38–43. doi: 10.1161/01.RES.78.1.38 8603503

[179] Mutgan AC Jandl K Kwapiszewska G. Endothelial basement membrane components and their products, matrikines: Active drivers of pulmonary hypertension? Cells (2020) 9(9):2029. doi: 10.3390/cells9092029 PMC7563239 32899187

[180] Bizbiz L Bonithon-Kopp C Ducimetiere P Berr C Alperovitch A Robert L. Relation of serum elastase activity to ultrasonographically assessed carotid artery wall lesions and cardiovascular risk factors. EVA study Atheroscl (1996) 120:47–55. doi: 10.1016/0021-9150(95)05676-9 8645370

[181] Yasmin McEniery CM Wallace S Dakham Z Pulsalkar P Maki-Petaja K. Matrix metalloproteinase-9 (MMP-9), MMP-2, and serum elastase activity are associated with systolic hypertension and arterial stiffness. Arterioscler Thromb Vasc Biol (2005) 25:372. doi: 10.1161/01.ATV.0000151373.33830.41 15556929

[182] Diaz-Canestro C Puspitasari YM Liberale L Guzik TJ Flammer AJ Bonetti NR. MMP-2 knockdown blunts age-dependent carotid stiffness by decreasing elastin degradation and augmenting eNOS activation. Cardiovasc Res (2021) 118 (10):2385–96. doi: 10.1093/eurheartj/ehab724.3378 34586381

[183] Xiong W MacTaggart J Knispel R Worth J Persidsky Y Baxter BT. Blocking TNF-alpha attenuates aneurysm formation in a murine model. J Immunol (2009) 183:2741–6. doi: 10.4049/jimmunol.0803164 PMC4028114 19620291

[184] Leopold JA. Vascular calcification: Mechanisms of vascular smooth muscle cell calcification. Trends Cardiovasc Med (2015) 25:267–74. doi: 10.1016/j.tcm.2014.10.021 PMC4414672 25435520

[185] Hayden MR Tyagi SC Kolb L Sowers JR Khanna R. Vascular ossification-calcification in metabolic syndrome, type 2 diabetes mellitus, chronic kidney disease, and calciphylaxis-calcific uremic arteriolopathy: The emerging role of sodium thiosulfate. Cardiovasc Diabetol (2005) 4:4. doi: 10.1186/1475-2840-4-4 15777477 PMC1079905

[186] Weng JJ Su Y. Nuclear matrix-targeting of the osteogenic factor Runx2 is essential for its recognition and activation of the alkaline phosphatase gene. Biochim Biophys Acta (2013) 1830:2839–52. doi: 10.1016/j.bbagen.2012.12.021 23287548

[187] Cui RR Li SJ Liu LJ Yi L Liang QH Zhu X. MicroRNA-204 regulates vascular smooth muscle cell calcification *in vitro* and *in vivo* . Cardiovasc Res (2012) 96:320–9. doi: 10.1093/cvr/cvs258 22871591

[188] Zhang J Zheng B Zhou PP Zhang RN He M Yang Z. Vascular calcification is coupled with phenotypic conversion of vascular smooth muscle cells through Klf5-mediated transactivation of the Runx2 promoter. Biosci Rep (2014) 34:e00148. doi: 10.1042/BSR20140103 25205373 PMC4219426

[189] Gutierrez FR Moran CJ Ludbrook PA McKnight RC Weldon CS. Pulmonary arterial calcification with reversible pulmonary hypertension. AJR Am J Roentgenol (1980) 135:177–8. doi: 10.2214/ajr.135.1.177 6771988

[190] Akmal M Barndt RR Ansari AN Mohler JG Massry SG. Excess PTH in CRF induces pulmonary calcification, pulmonary hypertension and right ventricular hypertrophy. Kidney Int (1995) 47:158–63. doi: 10.1038/ki.1995.18 7731141

[191] Ohyama Y Tsuchiya H Kurosawa K Nakano A Arai M Nobusawa S. Pulmonary hypertension with extensive calcification in small pulmonary vessels and alveolar capillary wall in a chronic hemodialysis patient. J Cardiol cases (2013) 8:e13–6. doi: 10.1016/j.jccase.2013.02.015 PMC6281483 30546730

[192] Aluja Jaramillo F Gutierrez FR Diaz Telli FG Yevenes Aravena S Javidan-Nejad C Bhalla S. Approach to pulmonary hypertension: From CT to clinical diagnosis. Radiographics (2018) 38:357–73. doi: 10.1148/rg.2018170046 29432063

[193] Tanguay VF Babin C Giardetti G Sohier-Poirier C Menard-Cholette V Ranchoux B. Enhanced pulmonary artery radiodensity in pulmonary arterial hypertension: A sign of early calcification? Am J Respir Crit Care Med (2019) 199:799–802. doi: 10.1164/rccm.201806-1027LE 30571924

[194] Philp T. Pulmonary artery calcification. Scott Med J (1972) 17:104–7. doi: 10.1177/003693307201700305 5057341

[195] Ma C Gu R Wang X He S Bai J Zhang L. circRNA CDR1as promotes pulmonary artery smooth muscle cell calcification by upregulating CAMK2D and CNN3 *via* sponging miR-7-5p. Mol Ther Nucleic Acids (2020) 22:530–41. doi: 10.1016/j.omtn.2020.09.018 PMC7566008 33230455

[196] Parhami F Basseri B Hwang J Tintut Y Demer LL. High-density lipoprotein regulates calcification of vascular cells. Circ Res (2002) 91:570–6. doi: 10.1161/01.RES.0000036607.05037.DA 12364384

[197] Paradise CR Galvan ML Kubrova E Bowden S Liu E Carstens MF. The epigenetic reader Brd4 is required for osteoblast differentiation. J Cell Physiol (2020) 235:5293–304. doi: 10.1002/jcp.29415 PMC8128078 31868237

[198] Sancisi V Manzotti G Gugnoni M Rossi T Gandolfi G Gobbi G. RUNX2 expression in thyroid and breast cancer requires the cooperation of three non-redundant enhancers under the control of BRD4 and c-JUN. Nucleic Acids Res (2017) 45:11249–67. doi: 10.1093/nar/gkx802 PMC5737559 28981843

[199] Meloche J Lampron MC Nadeau V Maltais M Potus F Lambert C. Implication of inflammation and epigenetic readers in coronary artery remodeling in patients with pulmonary arterial hypertension. Arterioscler Thromb Vasc Biol (2017) 37:1513–23. doi: 10.1161/ATVBAHA.117.309156 28473439

[200] Van der Feen DE Kurakula K Tremblay E Boucherat O Bossers GPL Szulcek R. Multicenter preclinical validation of BET inhibition for the treatment of pulmonary arterial hypertension. Am J Respir Crit Care Med (2019) 200:910–20. doi: 10.1164/rccm.201812-2275OC 31042405

[201] Watson KE Parhami F Shin V Demer LL. Fibronectin and collagen I matrixes promote calcification of vascular cells *in vitro*, whereas collagen IV matrix is inhibitory. Arterioscler Thromb Vasc Biol (1998) 18:1964–71. doi: 10.1161/01.ATV.18.12.1964 9848891

[202] Krohn JB Hutcheson JD Martinez-Martinez E Irvin WS Bouten CV Bertazzo S. Discoidin domain receptor-1 regulates calcific extracellular vesicle release in vascular smooth muscle cell fibrocalcific response *via* transforming growth factor-beta signaling. Arterioscler Thromb Vasc Biol (2016) 36:525–33. doi: 10.1161/ATVBAHA.115.307009 PMC4767541 26800565

[203] Hodroge A Trecherel E Cornu M Darwiche W Mansour A Ait-Mohand K. Oligogalacturonic acid inhibits vascular calcification by two mechanisms: Inhibition of vascular smooth muscle cell osteogenic conversion and interaction with collagen. Arterioscler Thromb Vasc Biol (2017) 37:1391–401. doi: 10.1161/ATVBAHA.117.309513 28522698

[204] Piera-Velazquez S Jimenez SA. Endothelial to mesenchymal transition: Role in physiology and in the pathogenesis of human diseases. Physiol Rev (2019) 99:1281–324. doi: 10.1152/physrev.00021.2018 PMC6734087 30864875

[205] Good RB Gilbane AJ Trinder SL Denton CP Coghlan G Abraham DJ. Endothelial to mesenchymal transition contributes to endothelial dysfunction in pulmonary arterial hypertension. Am J Pathol (2015) 185:1850–8. doi: 10.1016/j.ajpath.2015.03.019 25956031

[206] Li L Kim IK Chiasson V Chatterjee P Gupta S. NF-kappaB mediated miR-130a modulation in lung microvascular cell remodeling: Implication in pulmonary hypertension. Exp Cell Res (2017) 359:235–42. doi: 10.1016/j.yexcr.2017.07.024 28755990

[207] Suzuki T Carrier EJ Talati MH Rathinasabapathy A Chen X Nishimura R. Isolation and characterization of endothelial-to-mesenchymal transition cells in pulmonary arterial hypertension. Am J Physiol Lung Cell Mol Physiol (2018) 314:L118–26. doi: 10.1152/ajplung.00296.2017 PMC5866427 28935639

[208] Qiao L Nishimura T Shi L Sessions D Thrasher A Trudell JR. Endothelial fate mapping in mice with pulmonary hypertension. Circulation (2014) 129:692–703. doi: 10.1161/CIRCULATIONAHA.113.003734 24201301

[209] Crnkovic S Marsh LM El Agha E Voswinckel R Ghanim B Klepetko W. Resident cell lineages are preserved in pulmonary vascular remodeling. J Pathol (2018) 244:485–98. doi: 10.1002/path.5044 PMC5903372 29359814

[210] Dan Q Shi Y Rabani R Venugopal S Xiao J Anwer S. Claudin-2 suppresses GEF-H1, RHOA, and MRTF, thereby impacting proliferation and profibrotic phenotype of tubular cells. J Biol Chem (2019) 294:15446–65. doi: 10.1074/jbc.RA118.006484 PMC6802501 31481470

[211] Ranchoux B Antigny F Rucker-Martin C Hautefort A Pechoux C Bogaard HJ. Endothelial-to-mesenchymal transition in pulmonary hypertension. Circulation (2015) 131:1006–18. doi: 10.1161/CIRCULATIONAHA.114.008750 25593290

[212] Farrar EJ Hiriart E Mahmut A Jagla B Peal DS Milan DJ. OCT4-mediated inflammation induces cell reprogramming at the origin of cardiac valve development and calcification. Sci Adv (2021) 7:eabf7910. doi: 10.1126/sciadv.abf7910 34739324 PMC8570594

[213] Yao Y Jumabay M Ly A Radparvar M Cubberly MR Bostrom KI. A role for the endothelium in vascular calcification. Circ Res (2013) 113:495–504. doi: 10.1161/CIRCRESAHA.113.301792 23852538 PMC3851028

[214] Yao J Wu X Qiao X Zhang D Zhang L Ma JA. Shifting osteogenesis in vascular calcification. JCI Insight (2021) 6 (10):e143023. doi: 10.1172/jci.insight.143023 PMC8262274 33848269

[215] Xu D Li Y Zhang B Wang Y Liu Y Luo Y. Resveratrol alleviate hypoxic pulmonary hypertension *via* anti-inflammation and anti-oxidant pathways in rats. Int J Med Sci (2016) 13:942–54. doi: 10.7150/ijms.16810 PMC5165688 27994500

[216] Andreakos E. Targeting cytokines in autoimmunity: New approaches, new promise. Expert Opin Biol Ther (2003) 3:435–47. doi: 10.1517/14712598.3.3.435 12783612

[217] Hernandez-Sanchez J Harlow L Church C Gaine S Knightbridge E Bunclark K. Clinical trial protocol for TRANSFORM-UK: A therapeutic open-label study of tocilizumab in the treatment of pulmonary arterial hypertension. Pulm Circ (2018) 8:2045893217735820. doi: 10.1177/2045893217735820 28956500 PMC6852369

[218] Zamanian RT Badesch D Chung L Domsic RT Medsger T Pinckney A. Safety and efficacy of b-cell depletion with rituximab for the treatment of systemic sclerosis-associated pulmonary arterial hypertension: A multicenter, double-blind, randomized, placebo-controlled trial. Am J Respir Crit Care Med (2021) 204:209–21. doi: 10.1164/rccm.202009-3481OC PMC8650794 33651671

[219] Toshner M Church C Harbaum L Rhodes C Villar Moreschi SS Liley J. Mendelian randomisation and experimental medicine approaches to interleukin-6 as a drug target in pulmonary arterial hypertension. Eur Respir J (2022) 59(3):2002463. doi: 10.1183/13993003.02463-2020 PMC8907935 34588193

[220] Hudalla H Michael Z Christodoulou N Willis GR Fernandez-Gonzalez A Filatava EJ. Carbonic anhydrase inhibition ameliorates inflammation and experimental pulmonary hypertension. Am J Respir Cell Mol Biol (2019) 61:512–24. doi: 10.1165/rcmb.2018-0232OC PMC6775956 30951642

